# A New ternary organometallic Pd(ii)/Fe(iii)/Ru(iii) self-assembly monolayer: the essential ensemble synergistic for improving catalytic activity†

**DOI:** 10.1039/d0ra09347e

**Published:** 2021-01-04

**Authors:** Ruirui Ren, Pingping Huang, Wuduo Zhao, Tiesheng Li, Minghua Liu, Yangjie Wu

**Affiliations:** College of Chemistry and Molecular Engineering, Zhengzhou University Kexuedadao 100 Zhengzhou 450001 P. R. China lts34@zzu.edu.cn +86-371-67766667; Henan Institute of Advanced Technology, Zhengzhou University Kexuedadao 100 Zhengzhou 450001 Henan Province P. R. China; Beijing National Laboratory for Molecular Science, Institute of Chemistry, Chinese Academy of Sciences Zhongguancun North First Street 2 Beijing 100190 P. R. China

## Abstract

The synergistic catalytic effect in a hetero-trimetallic catalytic monolayer is one of the intriguing topics because the additive effects of the second or third component play an important role in improving the activity. In this paper, a new Schiff-base organometallic nanosheet containing Pd/Fe/Ru immobilized on graphene oxide (GO@H-Pd/Fe/Ru) was prepared and characterized. The catalytic performance of GO@H-Pd/Fe/Ru and synergistic effect were systematically investigated. GO@H-Pd/Fe/Ru was found to be an efficient catalyst with higher turnover frequency (TOF) (26 892 h^−1^) and stability with recyclability of at least 10 times in the Suzuki–Miyaura coupling reaction. The deactivation mechanism was caused by the aggregation of the active species, loss of the active species, the changes of the organometallic complex, and active sites covered by adsorbed elements during the catalytic process. GO@H-Pd/Fe/Ru was a heterogeneous catalyst, as confirmed by kinetic studies with *in situ* FT-IR, thermal filtration tests and poisoning tests. The real active center containing Pd, Ru and Fe arranged as Fe(iii)–Ru(iii)–Pd(ii)–Fe(iii) was proposed. Although Ru(iii) and Fe(iii) were shown to be less active or inactive, the addition of Fe and Ru could effectively improve the entire activity by their ‘‘indirect’’ function, in which Fe or Ru made Pd more negative and more stable. The ensemble synergistic effect between metals, the ligand and support was described as a process in which the electron was transferred from GO*via* ligand to Ru, and then to Pd or from Fe to Pd to make Pd more negative, promoting the oxidation addition with aryl halide. Also, the vicinity of Ru around Pd as the promoter adsorbed aryl boronic acid, which facilitates its synergism to react with the oxidation intermediate to the trans-metallic intermediate.

## Abstract 2

An ensemble synergistic effect between metals, ligand and support occurs when an electron transferred from GO*via* ligand to Ru, and then to Pd or from Fe to Pd, to make Pd more negative, resulting in higher activity.

## Graphical abstract

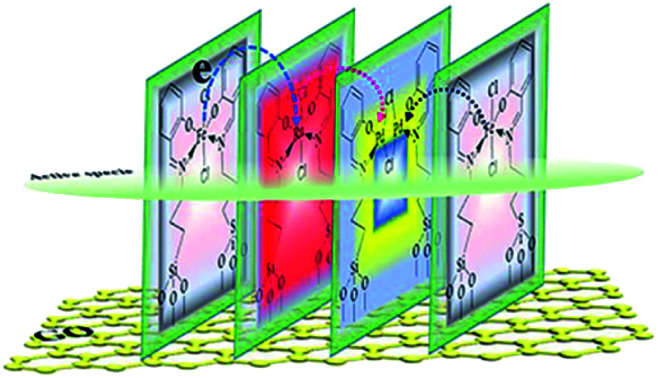

## 1. Introduction

The design and synthesis of heterogeneous catalysts are very important research areas, expanding from a mono- to multi-metallic catalyst, in which monometallic heterogeneous catalysts have been deeply studied.^1^ For hetero-bimetallic catalysts, an investigation on the relationship between their activity and structure is deeply studied for understanding the synergism between the different components.^2–4^ Recently, the synergistic catalysis with trimetallic catalysts have received considerable interest,^5^ and the real active centers remain unknown because each component is responsible for different steps in the overall transformation.^6^ Meanwhile, there has been rapidly growing interest in multi-component catalysts, in which improving the activity and reducing expenses are considered to be key issues for heterogeneous catalyst,^7,8^ and are particularly important for noble metals.^9–12^

In catalytic systems, homogeneous catalysis has some drawbacks, such as the problem of the residue and recycling of the catalyst.^13^ A heterogeneous catalyst immobilized or fixed on a support can be easily recycled.^14^ The heterogeneous Pd-catalyzed Suzuki cross-coupling reaction is a remarkably useful tool in organic synthesis.^15^ Therefore, substituting palladium with less expensive doped transition metals is expected for achieving higher activity and reducing the catalyst costs. For example, some economic transition metals include nickel with its charge-donating ability,^4,9^ and the charge transferring and stabilizing property of Fe.^8,16^ Ruthenium is one of the cheapest precious metals having broad application prospects,^17,18^ and has long been considered as one of the effective components in a catalyst.^19,20^ Synergetic effects with dopants for an efficient Pd-based, Fe-based and Ru-based heterogeneous bimetallic catalyst are intensively being investigated.^21–29^ If the multimetallic catalyst contains Pd, Fe and Ru or another component, they will show efficient synergism due to the consecutive effect of different active centers in reactions.^30–33^

The development of a multi-metallic catalyst is motivated by the idea to overcome the problems encountered in heterogeneous systems. The preparation of trimetallic catalysts, in which most of them are in the form of an alloy, involves the core–shell being synthesized by a complicated method and their reproducibility is difficult to control.^34^ Therefore, developing an efficient multi-metallic catalytic system must overcome more difficulties, including the preparation method, assembling each component to control the electronic effects between different metals, the distribution of active sites, and ensuring that they not only act as a promoter, but also provide stabilization for the active center. Meanwhile, the interaction between each metal and the overall performance impact are an urgent problem to be solved.^35^

It is well known that the catalytic surfaces are correlated to the activity. The precise surface modification by introducing proper metals or constructing proper morphology can easily tune the catalytic properties.^36,37^ The self-assembly (SA) was an efficient way to get the desired structure with special properties, which can offer the desired controllability of the orientation and stable monolayer.^38–45^ Previous studies showed that the activity of the bimetallic catalysts, or their monolayer and their recyclability, could be enhanced by tuning their orientation of the organometallic molecular, component, morphology and distributions of the active species, relating to the electrical characteristics of the supports, ligands, and synergism of the mono- or hetero-bimetallic catalytic films.^4,16,35,37,39,44,46–55^

By now, multicomponent catalysts have made great progress, such as multi-metallic NPs, who have more variables available for tuning their catalytic activity.^56^ Research on introducing a third or fourth metal to the ordered catalytic molecular monolayer is limited. It is urgently needed to focus on how to prepare ordered heterogeneous trimetallic catalytic films at the molecular level with the proper components, in addition to basic research on what factors affect the catalytic activity and rationality. Up to now, there have only been a few studies in the literature on the rational design in the directional order heterogeneous trimetallic catalytic monolayer.

Meanwhile, appropriate supports that improve the dispersion and the utilization of noble catalysts have attracted much attention. This is because the intrinsic activity and selectivity can vary strongly, depending on the physical properties of the supports.^57–60^ Graphene oxide as an efficient support was recognized as one of the ideal candidates because of its large surface area, high stability, unique two-dimensional structure.^61,62^ Thus, there are a variety of functional groups through which covalent self-assembly films can be anchored.^63–65^

Ligand design is also an integral part of the heterogeneous catalyst, and is promising for tuning the metal coordination sphere and stabilizing the active sites available for substrate binding during catalytic cycling.^66^ The Schiff-base ligand is a privileged ligand, which is capable of stabilizing many different metals and controlling the metal properties in many catalytic transformations.^67^

In this paper, an ordered hetero-trimetallic catalytic monolayer containing selected Pd,^68^ Fe^69^ and Ru^70^ metals coordinated with the Schiff-base ligand supported on GO by self-assembly was presented. It is expected to provide efficiency, recycling ability and a reduction in the overall economic cost. With the self-assembly method and the permutation of selecting the ligand, supports, and combination of different metals, the origin of the high activity and the relationship between the activity and the structure, and the synergism between the doped metals will be deeply explored and elucidated at molecular level using the Suzuki–Sonogashira coupling reaction as a template.

## 2. Experimental

### 2.1. Reagents and instruments, and the general procedure

Chemical reagents and instruments used for the characterization, general synthesis and general procedure for the coupling reaction and RactIR recording are presented in ESI.†

### 2.2. Preparation and characterization of the GO@H-Pd/Fe/Ru monolayer

**Scheme 1:**
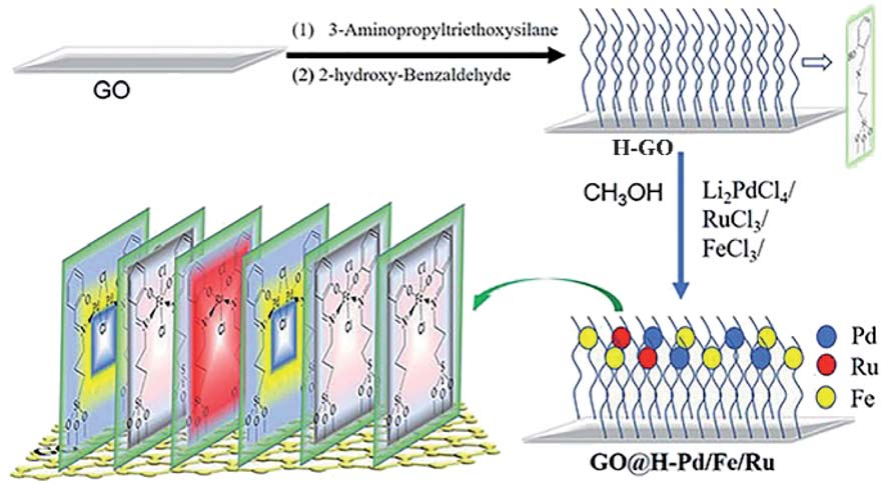
Preparation process of the GO@H-Pd/Fe/Ru monolayer.

The main target here was that the three different metals, having such a relation to each other, were arranged in a self-assembly monolayer that was fabricated as depicted in Scheme 1. The preparation processes of the GO@H-Pd/Fe/Ru monolayer were characterized by XRD, FT-IR, RS, SEM, TEM, XPS, ESI, and BEI (see ESI†).^16^

## 3. Results and discussion

### 3.1. Characterization of GO@H-Fe_95.73_Pd_4.26_Ru_0.01_

The XRD patterns of GO, H-GO and GO@H-Fe_95.73_Pd_4.26_Ru_0.01_ were measured (Fig. S1†). Two diffraction peaks were observed at 2*θ* values of 42.5° and 10.9°, corresponding to the (100) and (002) crystal planes of GO, respectively. In the case of H-GO and GO@H-Fe_95.73_Pd_4.26_Ru_0.01_, they shifted to a small angular direction compared to GO. According to the Bragg equation, 2*d* sin *θ* = *nλ*, the spacing of H-GO and GO@H-Fe_95.73_Pd_4.26_Ru_0.01_ increased due to the inserted ligand and organometallic compound.

From FT-IR (Fig. S2†), a peak could be observed due to the O–H group at 3430 cm^−1^. A peak at 1729 cm^−1^ corresponding to the stretching vibration of the carboxyl group, and three peaks at 1622 cm^−1^, 1221 cm^−1^, and 1053 cm^−1^ were the bending vibrations of C–OH and C–O on GO. After modification with the Schiff base, distinct peaks at 1105 and 1042 cm^−1^ corresponding to Si–O were observed. The stretching vibrational peak of C=N in the ligand at approximately 1636 cm^−1^ shifted to a lower frequency. This was due to the coordination of C=N with the Pd, Ru and Fe ions to form GO@H-Fe_95.73_Pa_4.26_Ru_0.01_.

The Raman spectra in the preparation process of GO@H-Fe_95.73_Pd_4.26_Ru_0.01_ are shown in Fig. S3,† which had two characteristic peaks at 1347 and 1596 cm^−1^ attributed to the D (C sp^2^) and G (C sp^3^) bands, respectively.^71^ The intensity ratio of *I*_D_/*I*_G_ increased from 0.95 to 0.97 and 0.99, which could be caused by two possible reasons. One reason was that some of the functional groups were reduced. The other was that the GO surface gradually became disordered during the functionalization process.

The chemical elements on the surface of GO@H-Fe_95.73_Pd_4.26_Ru_0.01_ obtained were measured by XPS (Fig. S4†). The energy level peaks of Pd 3d, C 1s, Si 2s, N 1s, O 1s, Fe 2p, Cl 2p, and Ru 3d were present in Fig. S4a.† The N 1s showed the characteristic peak at 400.0 eV in Fig. S4b.† The Ru 3d XPS spectrum of GO@H-Fe_95.73_Pd_4.26_Ru_0.01_ did not present the binding energies of Ru due to there being only a small amount of Ru, and quite close binding energies of C 1s (Fig. S4c†).^72^ There were two peaks with corresponding binding energies, 725.9 eV and 711.9 eV, of Fe 2p that were observed, as shown in Fig. S4d.† The binding energies, 343.6 eV and 338.6 eV, were denoted as the binding energy of Pd 3d, and showed that Pd(ii) existed in GO@H-Fe_95.73_Pd_4.26_Ru_0.01_ (Fig. S4e†).

The SEM images in the fabrication steps of GO@H-Fe_95.73_Pd_4.26_Ru_0.01_ were measured (Fig. S5†). Morphologies of GO exhibited the two-dimensional layered fold structures. After modification by the Schiff base ligand (H-GO), the layered structure showed more fold characteristics. The SEM image of GO@H-Fe_95.73_Pd_4.26_Ru_0.01_ showed a neat sheet structure, indicating the ordered modification of the Schiff-based complexes on graphene oxide.^73^ SEM-EDS of GO@H-Fe_95.73_Pd_4.26_Ru_0.01_ showed the presence of palladium, iron and ruthenium on the surface of the catalyst in Fig. S6.† This also indicated that the relative contents of different Pd, Fe and Ru metals were distributed on GO.

The TEM images in the preparation process of GO@H-Fe_95.73_Pd_4.26_Ru_0.01_ were obtained and are shown in Fig. S7.† A sheet-like structure of GO was observed (Fig. S7a†). Similar lamellar structures can be observed when the surface of GO was modified with a Schiff base, as shown in Fig. S7b.† From the TEM images of GO@H-Fe_95.73_Pd_4.26_Ru_0.01_, an obvious lamellar was observed (Fig. S7c†).

To further characterize the corresponding GO@H-Fe_95.73_Pd_4.26_Ru_0.01_ pore diameter distribution, BET was measured (Fig. S8†). GO, H-GO and GO@H-Fe_95.73_Pd_4.26_Ru_0.01_ isotherms were denoted to type IV, and GO@H-Fe_95.73_Pd_4.26_Ru_0.01_ was a H4 hysteresis loop (11.2948 m^2^ g^−1^). GO and H-GO were a H3 hysteresis loop (2.0953 m^2^ g^−1^, 4.8058 m2 g^−1^). GO@H-Fe_95.73_Pd_4.26_Ru_0.01_ was a mesoporous catalyst with pore sizes from 2 to 50 nm.^74^

EIS spectroscopy was widely utilized to investigate the electron transmission efficiency. The EIS diagrams for GO, H-GO and GO@H-Fe_95.73_Pd_4.26_Ru_0.01_ were measured (Fig. S9†). Theoretically, the arc in the EIS spectrum represents the impedance of the catalyst supported on the electrochemical nickel foam electrode. Smaller arcs represented faster electron transfer efficiency, and the diameter of the catalyst GO@H-Fe_95.73_Pd_4.26_Ru_0.01_ was smaller than that of GO and H-GO, indicating that GO@H-Fe_95.73_Pd_4.26_Ru_0.01_ showed a higher charge transfer capacity due to the proper arrangement of these three metallic complexes.^75^

For circumstantial evidence, the preparation process of the ligand monolayer and its complex monolayer on hydrophilic silicon by SAMs was characterized by AFM. AFM images of the prepared Si@OH, Si@H, and Si@H-Fe/Pd/Ru are presented in Fig. S10a–c.† The images showed the regular structures associated with different roughnesses in different steps, and the roughness was 0.45 nm, 1.13 nm, and 0.80 nm, respectively. It was evidence of the ordered arrangement of the molecules in the monolayer during SAMs, indicating that uniform monolayers were formed.

All characterizations obtained above provided evidence that the GO@H-Fe_95.73_Pd_4.26_Ru_0.01_ monolayer on the surface of GO was fabricated.

### 3.2. Evolution of the catalytic properties of GO@H-Fe_95.73_Pd_4.26_Ru_0.01_

#### 3.2.1 Optimization for the Suzuki coupling reaction

The catalytic performance of GO@H-Fe_95.73_Pd_4.26_Ru_0.01_ for the Suzuki coupling reaction was investigated (Table S1,† entries 1–15). Decreasing the reaction time, reducing the reaction temperature, and increasing the substrate amount resulted in a decrease in the reaction yield (entries 16–19). Under the reaction conditions (80 °C, 1 h, water–ethanol ratio of 3 : 1, Na_2_CO_3_), 99% yield of 4-phenyltoluene was obtained. The optimized reaction conditions were selected, as indicated in the following experimental section (entry 7).

#### 3.2.2 Effect of the metal ratio (Fe/Pd/Ru) on the catalytic activity

Different ratios of Fe/Pd/Ru in the catalyst were used to investigate the catalytic activity under the optimized reaction conditions shown in Table S2.† A yield of 99% was obtained for GO@H-Fe_95.73_Pd_4.26_Ru_0.01_, and the TOF was 17 247 (entry 9). Although the TOF of GO@H-Fe_95.73_Pd_4.26_Ru_0.01_ was 21 714, the yield was only 76% (entry 10). In the case of other bimetallic catalysts (entries 1–8), lower TOF values were obtained compared with that of GO@H-Fe_95.73_Pd_4.26_Ru_0.01_, in which it showed that the doped Fe and Ru could further enhance the catalytic activity.

Results showed that the trimetallic GO@H-Fe_95.73_Pd_4.26_Ru_0.01_ monolayer with proper ratio exhibited better catalytic activity than others, indicating that the concerted electronic effects between these three metals exhibited a positive synergism.^30^ Compared to GO@H-Pd, GO@H-Fe, GO@H-Ru, GO@H-Fe/Ru, GO@H-Pd/Fe and the GO@H-Pd/Ru monolayer (Table S2,† entries 1–6), the GO@H-Pd/Fe/Ru monolayer showed higher activity and stability, while preserving the beneficial catalytic activity of the Pd metallic monolayer (entries 7–10). The strategy revealed the great potential in the fundamental studies and preparation of the novel catalyst by utilizing the multimetallic monolayer. Considering the cost and environmental requirements, GO@H-Fe_95.73_Pd_4.26_Ru_0.01_ was selected for the following investigation on the catalytic properties.

#### 3.2.3 Catalytic properties of GO@H-Fe_95.73_Pd_4.26_Ru_0.01_

The control experiments were designed to explore the influence of the carrier, ligand, and preparation methods on the catalytic performance. There were no products with GO or H-GO (Table S3,† entries 1 and 2). The effect of the added GO was studied (entries 3–6). Li_2_PdCl_4_ (0.00287% mmol)/FeCl_3_·6H_2_O/RuCl_3_·*x*H_2_O added to the reaction mixture gave 84% yield (entry 3). When a mixture of Schiff base ligands and Li_2_PdCl_4_ (0.00287% mmol)/FeCl_3_·6H_2_O/RuCl_3_·*x*H_2_O were used as catalyst, only 42% yield was obtained (entry 4). The yield of GO/Li_2_PdCl_4_/FeCl_3_·6H_2_O/RuCl_3_·*x*H_2_O was 64% (entry 5). However, the yield of GO/Ligand/Li_2_PdCl_4_/FeCl_3_·6H_2_O/RuCl_3_·*x*H_2_O was only 49% (entry 6). In the case of (entry 7), higher yield and TOF values were obtained, showing that the ordered self-assembly metallic monolayer was an efficient catalyst (entry 7). Using silica as a support gave a better yield (entry 8), but it was lower than that of GO@H-Fe_95.73_Pd_4.26_Ru_0.01_, indicating that GO played a great role for enhancing the activity.

The catalytic activities of GO@H-Fe_95.73_Pd_4.26_Ru_0.01_ compared with other reported nanoporous catalysts are summarized in Table S4.†GO@H-Fe_95.73_Pd_4.26_Ru_0.01_ had a lower loading and a higher TOF value.

To investigate the scope of the substrates, the Suzuki–Sonogashira coupling reactions catalyzed by GO@H-Fe_95.73_Pd_4.26_Ru_0.01_ were conducted by reacting aryl halides with aryl boric acid derivatives (Table S5†). When aryl iodine and benzene boric acids were used, 99% yield was obtained (Table S5,† entries 1 and 2). When aryl bromides having electron-donor or electron-receptor functional groups and benzene boric acids were used, higher yields were obtained (Table S5,† entries 3–13). The different substitution groups of aryl bromides were compared, and the yield of *para*-substituted or *meta*-substituted were higher than that of *ortho*-substituted aryl bromides due to the steric hindrance (Table S5,† entries 8–13). However, in the case of the chlorobenzene used, the results were unsatisfactory. In addition, the phenylboronic derivatives showed higher reactivity than heterocyclic borate. It was the fact that the heteroatoms may coordinate with the active metal centers, resulting in the deactivation of the active metal.

#### 3.2.4 Recycling and stability of GO@H-Fe_95.73_Pd_4.26_Ru_0.01_

**Fig. 1:**
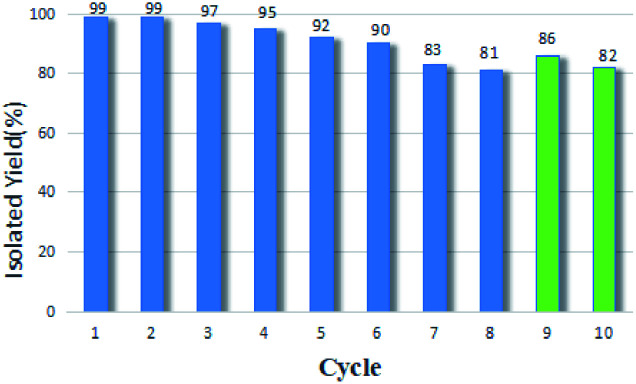
The recycle experiments of GO@H-Fe_95.73_Pd_4.26_Ru_0.01_.

In order to study the reusability of GO@H-Fe_95.73_Pd_4.26_Ru_0.01_, the recycling experiments were carried out in the optimized condition. As shown in Fig. 1, GO@H-Fe_95.73_Pd_4.26_Ru_0.01_ showed excellent stability upon reuse, which was better than that of the GO@H-Pd/Fe monolayer.^16^ After eight cycles, the catalyst maintained its high efficiency without causing significant loss. By extending the catalytic time to two hours, the yield was 82% at the 10th recycle. It was attributed to the loss of the active centers, according to the results of the amount of metals measured by ICP-AES before and after being recycled. The amount of metals was Pd: 3.31 × 10^−6^ mmol mg^−1^ (fresh sample: 2.87 × 10^−5^), Fe: 1.48 × 10^−6^ mmol mg^−1^ (fresh sample: 7.98 × 10^−5^), Ru: 4.79 × 10^−7^ mmol mg^−1^ (fresh sample: 8.70 × 10^−7^) at the 10th recycle.

#### 3.2.5 Investigation of the deactivation mechanism of GO@H-Fe_95.73_Pd_4.26_Ru_0.01_

It was important to understand the deactivation mechanism for the heterogeneous catalysts. To clarify the deactivation mechanism of GO@H-Fe_95.73_Pd_4.26_Ru_0.01_, TEM, SEM, XPS, and the content of metals in the catalyst before and after it was recycled were used to closely investigate the catalyst.^76^

First, the amount of metals was Pd: 3.31 × 10^−6^ mmol mg^−1^ (fresh sample: 2.87 × 10^−5^), Fe: 1.48 × 10^−6^ mmol mg^−1^ (fresh sample: 7.98 × 10^−5^), Ru: 4.79 × 10^−7^ mmol mg^−1^ (fresh sample: 8.70 × 10^−7^) at the 10th recycle measured by ICP-AES. The loss of the active centers was one reason for the deactivation of the catalyst, according to the results of the amount of metals in the catalyst before and after being recycled.

**Fig. 2:**
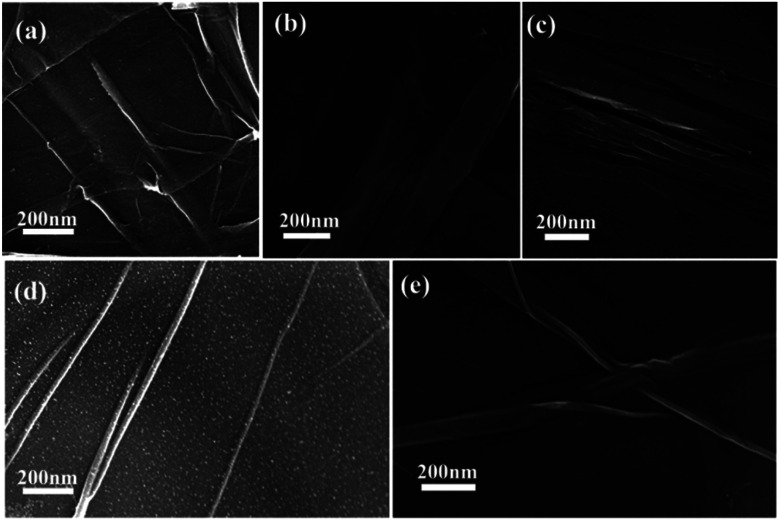
SEM images of the process of the catalyst and reused catalyst: (a) 0 min, (b) after the 1st run, (c) after the 4th run, (d) after the 8th run, and (e) after the 10th run.

**Fig. 3:**
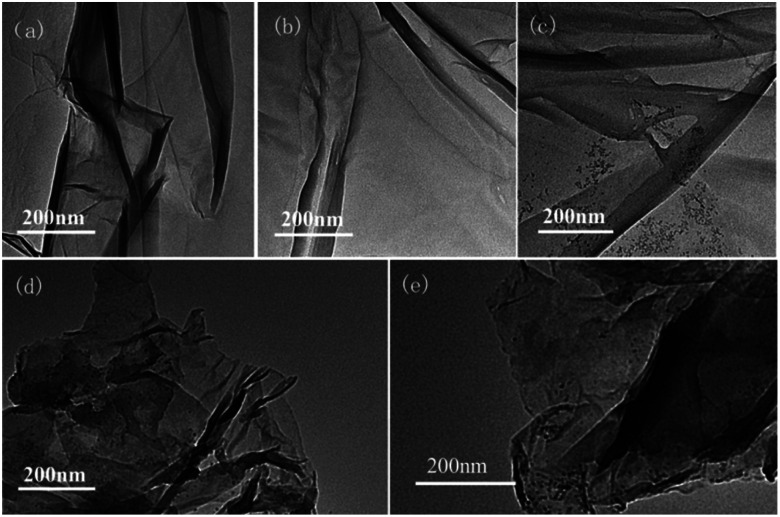
TEM images of the process of the catalyst and reused catalyst: (a) 0 min, (b) after the 1st run, (c) after the 4th run, (d) after the 8th run, and (e) after the 10th run.

**Fig. 4:**
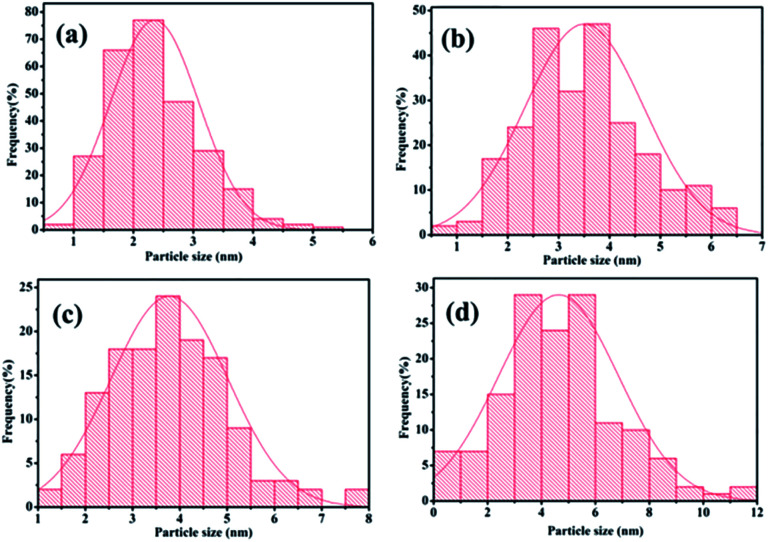
Histogram of the Pd(0) nano-cluster diameters during the catalytic process from Fig. 3: (a) 2.35 nm after the 1st run, (b) 3.50 nm after the 4th run, (c) 3.78 nm after the 8th run, (d) 4.32 nm after the 10th run.

Second, the catalyst morphology before and after being used was measured with SEM, as shown in Fig. 2, in which the morphology and structure remained the original two-dimensional layered structure during the reaction and after the 10th recycle. The TEM images of GO@H-Fe_95.73_Pd_4.26_Ru_0.01_ are shown in Fig. 3, from which a histogram of the Pd(0) diameters during the catalytic process was plotted (Fig. 4). Obviously, a slight agglomeration with the increased size of the catalyst could be observed after the fourth, eighth, and tenth cycles, meaning that deactivation of the activity was clearly induced by a slight agglomeration of the active species.^77^

The analysis by TEM and SEM showed that GO@H-Fe_95.73_Pd_4.26_Ru_0.01_ maintained better stability during recycling. In fact, the heterogeneous surface contained a few active sites, and the changes of the surface environment could affect the catalyst activity.^78^ It also indicated that the trimetallic complex monolayer acting as a pre-catalyst was likely a “reservoir” of the highly reactive species during the catalytic recycling.^6^

**Fig. 5:**
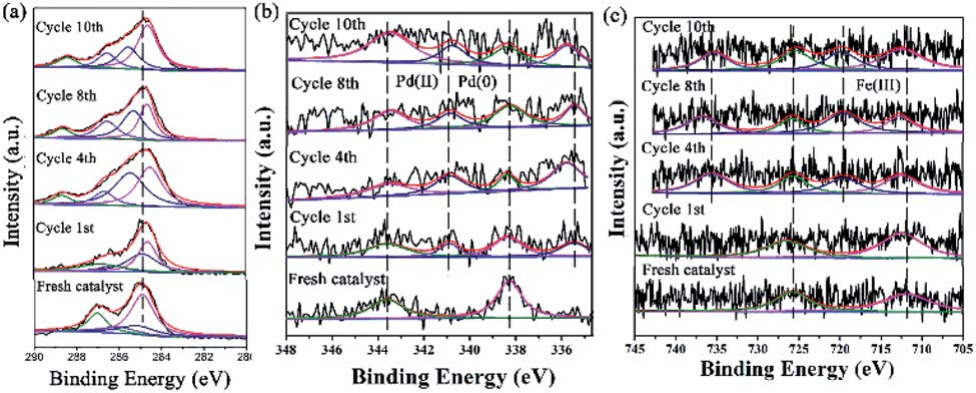
The changes of the HR-XPS spectra of GO@H-Pd/Fe/Ru before and after being recycled. (a) C 1s + Ru 3d; (b) Pd 3d; (c) Fe 2p.

XPS is a versatile surface analysis technique that can be used for examining the compositional and chemical states in the catalyst. The XPS spectra of the catalyst monolayers were measured in order to understand what happened for the structure and composition on the catalytic surface before and after being recycled (Fig. 5).

Fig. 5a showed two peaks centered at 284.95 and 287.05 eV, respectively. It has been demonstrated that the Ru(0) and Ru(ii) 3d_5/2_ binding energies were not detected because of the low amount of Ru.^72^ The HR spectrum of C 1s was deconvoluted into four components, with the component at 284.78 eV (C–C from GO) shifting to lower BE, and 285.12 eV (C=C, derived from GO and ligand) shifting to higher BE, showing that the GO acted as an electron donor. The BE peak at 285.95 eV corresponded to the C–O group, and the 287.05 eV peak attributed to the –C=O group shifted to higher BE, meaning that the structure changed during the catalytic process (Table S6†). The BE peaks of Pd 3d centered at 343.60 and 338.60 eV designated to Pd(ii) were present before cycling (Fig. 5b). Two new peaks appeared at 340.88 and 335.49 eV (denoted as Pd^0^) after the 1st recycle, indicating that Pd^0^ was formed during catalysis (Table S7†). The ratio of Pd^0^/Pd^2+^ increased with increasing recycling times (Table S8†), implying that more Pd^0^ appeared and resulted in an increased aggregation, which resulted in the deactivation of the catalyst. It was consistent with the results obtained from TEM (Fig. 3 and 4).

In the case of Fe(iii), the Fe 2p BE exhibited stability after the first run and a satellite peak appeared after the 2nd run, showing that the valence of Fe^3+^ increased (Table S7†). This enables the formation of Fe^*δ*+^, from which the electrons were transferred from Fe to Pd, resulting in the synergism between the metals to improve the activity (Fig. 5c).

**Fig. 6:**
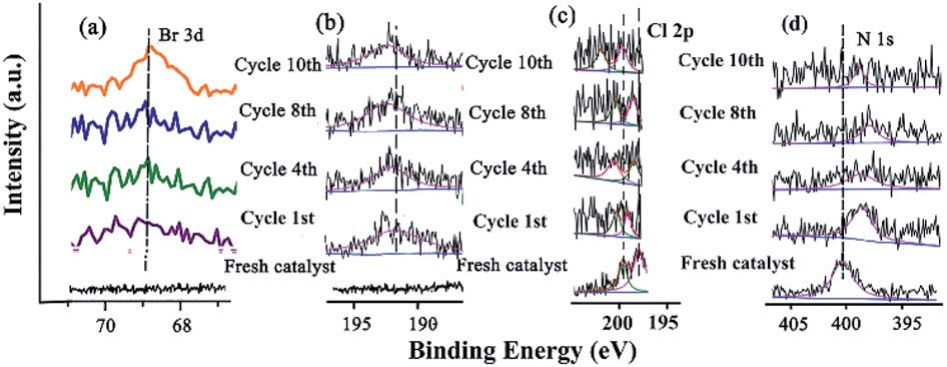
XPS spectra of (a) Br 3d, (b) B 1s, (c) Cl 2p, and (d) N 1s before and after being recycled.

The changes of the base elements on the surface of the catalyst were also investigated before and after being recycled (Fig. 6). There was a little residue of Br 3d and B 1s after being recycled, compared with that before recycle (Fig. 6a and b), indicating that these two elements might cover some active site after several reuse cycles, resulting in the deactivation of the catalyst. The Cl 2p peaks at 198.08 eV and 199.82 eV shifted to higher BE after catalysis (Fig. 6c and Table S9†), meaning that Cl coordinated with Pd and Pd^2+^. However, the peak of N 1s (the C=N double bond) at 401.51 eV shifted to lower BE after being recycled several times (Fig. 6d and Table S9†), indicating that the precursor of the Schiff-based Pd(ii) produced more active Pd(0) and a more available Schiff-base group appeared after more recycling. This free Schiff-base group could also stabilize and prevent Pd(0) from aggregating and leaching during the reaction.^79^ Meanwhile, the coordination of the C=N double bond of the ligand to the Pd(0) site brings the C–Br bond close to the Pd active site, which makes the oxidation easy due to the nature of the ligands around a redox-active metal center.

**Fig. 7:**
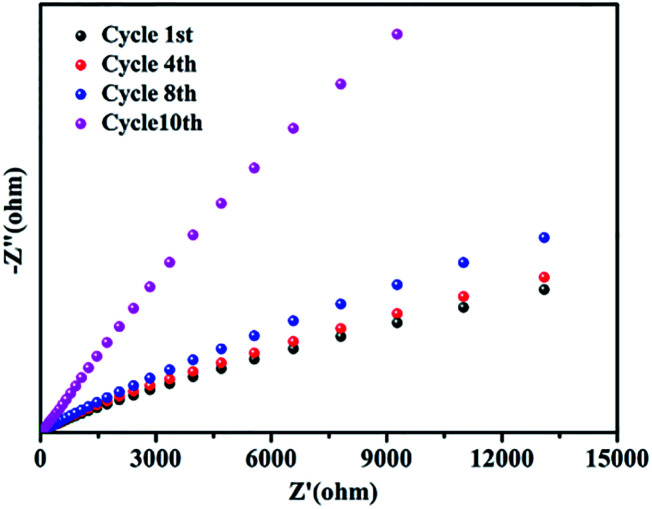
The changes of the EIS of GO@H-Fe_95.73_Pd_4.26_Ru_0.01_ in the recycling experiments.

The stability of GO@H-Pd/Fe/Ru after being recycled was further investigated by electrochemical impedance spectroscopy (EIS).^72^ No obvious changes of the resistance were observed before the 8th recycling (Fig. 7), indicating the better stability of the catalyst. However, the charge transfer resistance was significantly increased in the 10th cycling due to the change of the structure of the metallic complexes in the monolayer, residue of substrates adsorbed, and increased formation of zero-palladium on the surface of the monolayer, resulting in the deactivation of the catalyst.

According to the results obtained above for the recycled catalyst characterized by the amount of metals in the catalyst, SEM, TEM, XPS, and EIS, the inactivation mechanism of the catalyst mainly was the aggregation of the active species, leaching of the active species, the changes of the organometallic ligand, and the covered active sites by the adsorbed elements during the recycling process.

### 3.3. Investigation of the catalytic mechanism

#### 3.3.1 Kinetic, hot filtration and poisoning experiments

It was a significant problem of how to identify the heterogeneous catalysis, by which elucidating the catalytic mechanism can be achieved. It was very helpful to solve this problem through kinetics, hot filtration and poisoning tests.^80^

The yield increased rapidly in the earlier stage (before 35 minutes), and then slowly and up to 99% in 60 min (Fig. S11,† black line). To study whether the leaching of the active species occurred during the catalytic process, GO@H-Fe_95.73_Pd_4.26_Ru_0.01_ was removed after 15 min, and the reaction kinetics was measured from then on. The output did not increase and basically retained the same yields (Fig. S11,† red line), indicating that there was almost no leaching during catalysis.^81^

To further verify whether or not catalysis proceeded on the catalyst surface, poisoning experiments were carried out (Table S10†). The catalytic activity was not completely inhibited when a small amount of mercury was added to the reaction system. Mercury could not fully contact the active site of the catalyst because of the poor dispersion of mercury, and the active sites on the catalyst surface could not be completely poisoned. When 2′2-dipyridyl was added, the activity of the catalyst significantly decreased and was even almost completely deactivated, which indicated that the catalytic active sites were on the surface of GO@H-Fe_95.73_Pd_4.26_Ru_0.01_. When thiophene additives were used to poison the catalyst, a yield of only 6% was obtained. Therefore, the catalytic process occurring on the surface of GO@H-Fe_95.73_Pd_4.26_Ru_0.01_ could be determined.^82^

**Fig. 8:**
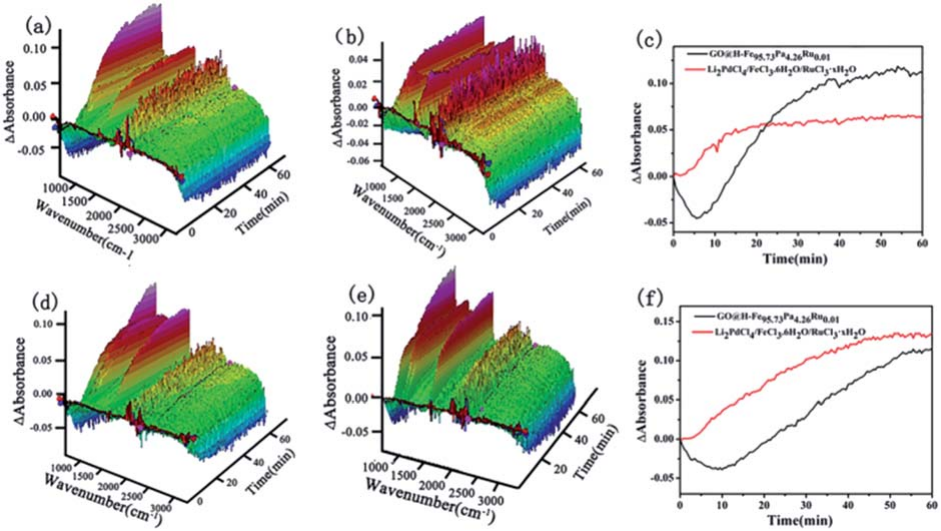
ReactIR plots over time. (a) 3D map, GO@H-Fe_95.73_Pd_4.26_Ru_0.01_ at 80 °C, (b) 3D map, Li_2_PdCl_4_/FeCl_3_·6H_2_O/RuCl_3_·*x*H_2_O at 80 °C, (c) kinetic curves of the catalytic reactions of GO@H-Fe_95.73_Pd_4.26_Ru_0.01_ and Li_2_PdCl_4_/FeCl_3_·6H_2_O/RuCl_3_·*x*H_2_O using the bands at 754 cm^−1^ from (a) and (b) at 80 °C. (d) 3D map, GO@H-Fe_95.73_Pd_4.26_Ru_0.01_ at 65 °C, (e) 3D map, Li_2_PdCl_4_/FeCl_3_·6H_2_O/RuCl_3_·*x*H_2_O at 65 °C, (f) kinetic curves of catalytic reactions of GO@H-Fe_95.73_Pd_4.26_Ru_0.01_ and Li_2_PdCl_4_/FeCl_3_·6H_2_O/RuCl_3_·*x*H_2_O using the bands at 754 cm^−1^ from (d) and (e) at 65 °C.

*In situ* ReactIR is usually used to investigate the catalytic process. ReactIR of the reactions catalyzed by GO@H-Fe_95.73_Pd_4.26_Ru_0.01_ and Li_2_PdCl_4_/FeCl_3_·6H_2_O/RuCl_3_·*x*H_2_O at different temperatures were measured, as shown in Fig. 8. For GO@H-Fe_95.73_Pd_4.26_Ru_0.01_, the peak intensities at 754 nm increased with increasing time at different temperatures (Fig. 8a and d), and had the S-type curve with an “induction period” (Fig. 8c and f, black line), which was the characteristic of the heterogeneous catalysis. However, in the case of Li_2_PdCl_4_/FeCl_3_·6H_2_O/RuCl_3_·*x*H_2_O, the induction period could not be observed (Fig. 8b and e; red line), indicating the difference in the catalytic mechanism for GO@H-Fe_95.73_Pd_4.26_Ru_0.01_. According to the results of GO@H-Fe_95.73_Pd_4.26_Ru_0.01_, the active species formed before catalysis related to the formation of the Pd nanoparticles, possibly Ru, and any other transition-metal nanocluster systems.^83^ Then, the substrates adsorbed on the catalytic surface made contact with the active center to form intermediates, followed by the generation of products, and the desorption from the surface.^84^ The results obtained by the kinetic studies, hot filtration and poisoning experiments were evidence that this catalysis was a heterogeneous catalysis.

**Fig. 9:**
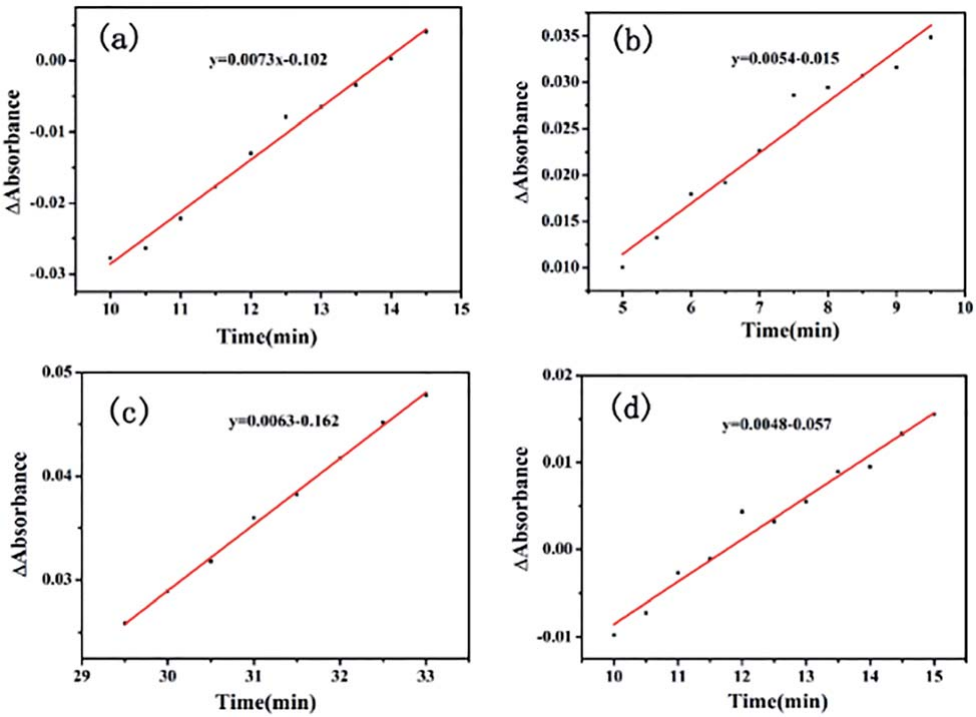
ReactIR plots over time. (a) GO@H-Fe_95.73_Pd_4.26_Ru_0.01_ using the band at 754 cm^−1^ at 80 °C, (b) Li_2_PdCl_4_/FeCl_3_·6H_2_O/RuCl_3_·*x*H_2_O at 80 °C. (c) GO@H-Fe_95.73_Pd_4.26_Ru_0.01_ using the band at 754 cm^−1^ at 65 °C, (d) catalyzed byLi_2_PdCl_4_/FeCl_3_·6H_2_O/RuCl_3_·*x*H_2_O at 65 °C.

The *E*_a_ value of the heterogeneous catalysis was 9.70 kJ mol^−1^ (*k*_1_ = 0.0073, 80 °C; *k*_2_ = 0.0063, 65 °C) from Fig. 9a and c. Under the same conditions, the apparent activation energy of the homogeneous Li_2_PdCl_4_/FeCl_3_·6H_2_O/RuCl_3_·*x*H_2_O was 7.9 kJ mol^−1^ (*k*_1_ = 0.0054, 80 °C; *k*_2_ = 0.0048, 65 °C) from Fig. 9b and d obtained by the Arrhenius formula *k* = *A* × exp(*E*_a_/*RT*).

The results obtained seem contradictory since the *E*_a_ (Homo) value was lower than that of *E*_a_ (Hetero). To investigate whether the supports used affect the heterogeneous reaction, the same amount of graphene oxide (GO), carbon power (CP) or Silica was applied to the homogeneous Li_2_PdCl_4_/FeCl_3_·6H_2_O/RuCl_3_·*x*H_2_O, respectively.^85^ The dynamic curves of Li_2_PdCl_4_/FeCl_3_·6H_2_O/RuCl_3_·*x*H_2_O with GO, CP or Silica were measured as shown in Fig. S12–S14,† and the apparent activation energies are listed in Table S11.† These apparent activation energies were higher than that of GO@H-Fe_95.73_Pd_4.26_Ru_0.01_, indicating that the ordered heterogeneous trimetallic catalytic monolayer supported on GO had higher catalytic activity than those of Li_2_PdCl_4_/FeCl_3_·6H_2_O/RuCl_3_·*x*H_2_O mixed with GO, CP and Silica.

#### 3.3.2 Investigation on the electrochemical impedance spectra (EIS) during catalysis

The electrochemical impedance spectroscopy (EIS) changes were also utilized for exploring what happened during the reaction process (Fig. S15†). The diameter variation of EIS was incremental with increasing times, and the charge transfer resistances of GO@H-Pd/Fe/Ru increased after catalysis. It was speculated that the reduced Pd^0^ during the reaction made the charge transfer difficult, resulting in higher resistance. It also indicated that the stability of the used GO@H-Pd/Fe/Ru became lower than that of the fresh GO@H-Pd/Fe/Ru.^72^

#### 3.3.3 Investigation of the catalytic process

##### 3.3.3.1 Raman, SEM and TEM analysis on the catalytic process

The Raman spectra of GO@H-Fe_95.73_Pd_4.26_Ru_0.01_ during the reaction process are shown in Fig. S16.†GO@H-Fe_95.73_Pd_4.26_Ru_0.01_ still maintained the two characteristic D and G bands during the reaction. The shift of the two peaks was not observed during the reaction, indicating the stability of the catalyst. Compared with fresh GO@H-Fe_95.73_Pd_4.26_Ru_0.01_, the recovered GO@H-Fe_95.73_Pd_4.26_Ru_0.01_ showed a higher *I*_D_/*I*_G_ strength ratio due to the micro-environment change on the surface of the catalyst.^86^ To investigate the morphology of the surface during the catalytic process, SEM images were measured at 0 min, 10 min, 40 min and 60 min (Fig. S17a–d†). It could be seen from the images that the morphology of the GO@H-Pd/Fe/Ru monolayer had no changes at different reaction times, indicating that the catalyst still maintained the original state during the reaction process. In the case of the TEM of catalyst (Fig. S18†), almost no metal particles were observed (Fig. S17a–d†) when finished in 1 h, implying a higher stability of the catalyst.

##### 3.3.3.2 Investigation on the arrangement of the multi-metallic catalytic monolayer

X-ray photoelectron spectroscopy (XPS) is an effective surface analysis technique to investigate the elements in the trimetallic catalytic monolayer, and is informative enough for exploring the real active center during the catalytic process,^87^ which is dominated mainly by the metal composition on the surface and structure of the catalytic monolayer. By elucidating the true species, including its change in catalytic monolayer, the catalytic mechanism could be proposed.

**Fig. 10:**
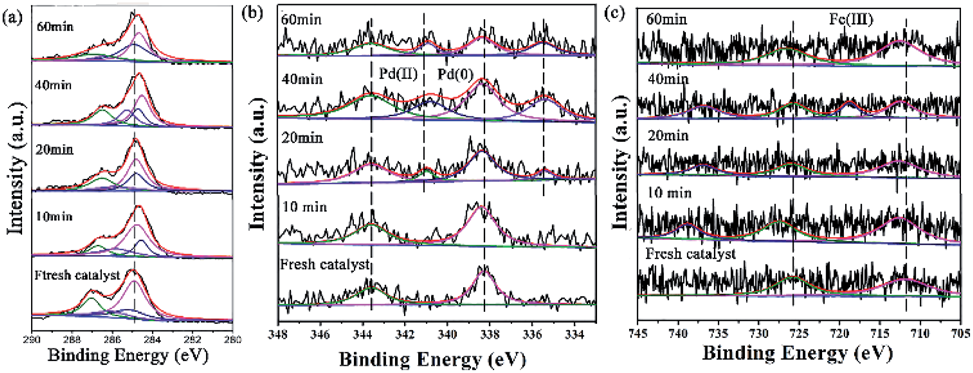
XPS images of GO@H-Pd/Fe/Ru in the catalytic process and the recycled catalyst: (a) C 1s + Ru 3d, (b) Pd 3d, (c) Fe 2p.

Peaks of Ru at 279.80 eV, 280.8 eV were ambiguous due to the overlap with C 1s and too low of a Ru quantity or low Ru content to be observed in this experiment (Fig. 10a).^88^ On the other hand, the peak at 284.81 eV was attributed to the C=C group of GO. The peak at 285.92 eV was the C=N group in the ligand, and the peaks at 286.25 and 288.16 eV were attributed to COOH and C–OH, respectively. The data are listed in Table S12.†

For GO@H-Fe_95.73_Pd_4.26_Ru_0.01_, the Pd^2+^ species existed at an early stage. After 20 minutes of reaction, a pair of peaks appeared at 340.65 and 335.40 eV, corresponding to Pd 3d 3/2 and 3/5 (Pd^0^) (Fig. 10b), indicating that the active Pd^0^ was formed at the beginning of the catalytic reaction.^89^ The intensity of the Pd^0^ peak increased with increasing reaction time compared to that of Pd^2+^, which also proved that Pd^2+^ was reduced to Pd^0^ during the catalytic reaction. The deconvolution data are presented in Table S13.†

The XPS spectrum of Fe 2p displayed two characteristic peaks of Fe^3+^ at 711.90 and 725.70 eV (Fig. 10c). Compared with the fresh catalysts, the binding energy of Fe^3+^ during catalysis moved towards higher binding energy. In addition, a satellite peak for Fe^3+^ at a higher BE (738.79 eV), which was very sensitive to the oxidation states, appeared after 10 min. Two additional satellite peaks for Fe^3+^ at a higher BE (718.65, 738.79 eV), which also was very sensitive to the oxidation states, appeared after 40 min and recovered after the reaction finished, meaning that Fe^3+^ was the electron donor during the catalytic process.^90^ The deconvolution data are presented in Table S13.†

**Fig. 11:**
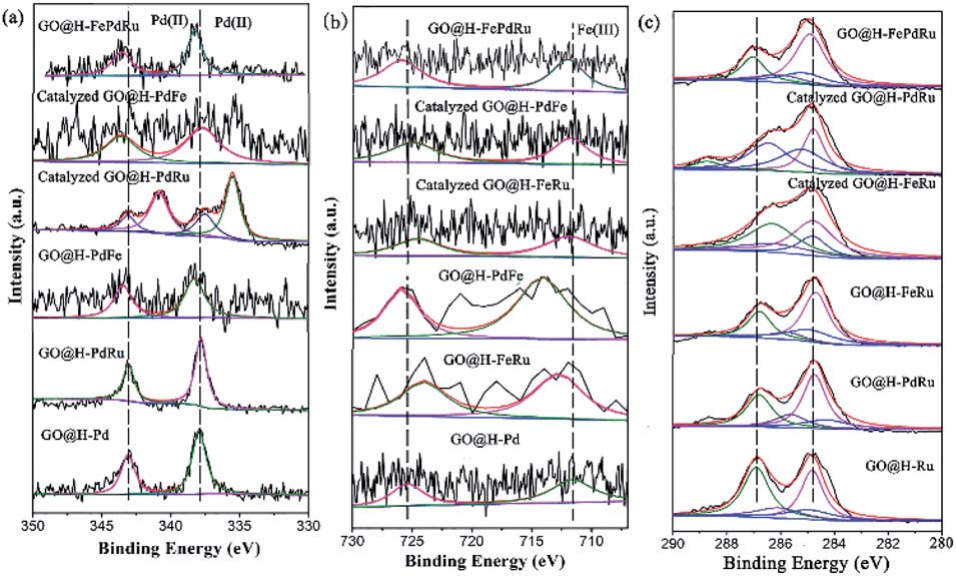
XPS analysis for GO@H-Pd/Fe/Ru, Pd/Ru, Pd/Fe and Ru/Fe monolayer before and after catalysis.

Considering that the combination of different metals in the trimetallic monolayer has an important role in catalysis, it is plausible to propose that the proper arrangement in the multimetallic monolayer can be beneficial in enhancing the catalytic activity and stability, by bringing them together in a controlled manner. Therefore, the XPS analysis for the Pd/Ru, Pd/Fe and Ru/Fe monolayer used for catalysis were investigated in detail, as shown in Fig. 11. It also has to be mentioned, however, that a relatively small amount of Ru (0.01%) was present, the analyses of which are relatively difficult. Therefore, these spectra were not deconvoluted.

As is know, high activity is attributed to the sequential electronic effect among the different metals in the monolayer, and also to the electronic effect between the ligands and metals. This concept can be applied to the trimetallic nanosheet prepared, although it was hard to predict the preferable position of the Pd(ii), Fe(iii) and Ru(iii) ions in the monolayer.^91^ The deconvolution data for Pd 3d and Fe 2p of the mono-, bi-, and trimetallic catalysts before and after being used for catalysis are listed in Table 1.

**Table 1:** Deconvolution data for Pd 3d and Fe 2p of different catalysts before and after being used

No.	Catalyst	Pd 3d BE (eV)		Fe 2p BE (eV)	
1	GO@H-Pd	343.09	337.94		
2	GO@H-PdRu	343.04	337.80		
3	GO@H-PdFe	343.43	338.25	714.14	725.90
4	GO@H-FeRu			712.74	724.02
5	GO@H-Ru				
6	GO@H-Fe			711.60	725.40
7	Catalyzed GO@H-PdRu	343.09	337.50		
8	Catalyzed GO@H-PdFe	343.58	337.70	711.80	725.00
9	Catalyzed GO@H-FeRu			711.88	724.60
10	GO@H-PdFeRu	343.60	338.20	711.90	725.70
11	Catalyzed GO@H-PdFeRu	343.61	338.33	712.33	726.25

The BE of Pd(ii) varied with various combinations, as shown in Fig. 11a. The results showed that the BE of Pd(ii) shifted to lower BE after catalysis (Table 1, entries 2, 3, 7, 8, 10 and 11), implying that both Fe(iii) and Ru(iii) were electron donors, which could make Pd(ii) more negative than boost the activity.

The BE of Fe(iii) varied with various combinations, as shown in Fig. 11b. The results showed that the BE of Fe(iii) shifted to higher BE during the catalytic process, implying that Fe(iii) mainly was an electron donor that could make Pd(ii) and Ru(iii) more negative than it could boost the activity (Table 1, entries 3, 4, 6, 8, 9, 10 and 11). The similar result for Ru showed that the catalyzed GO@H-Pd/Ru gave Pd^0^ with a ratio of (Pd^0^/Pd^2+^ = 1.38), indicating that Ru acted as a donor to make Pd more negative.

**Scheme 2:**
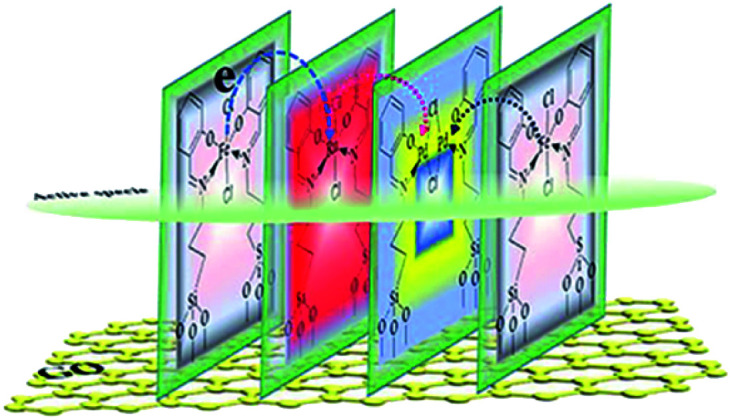
Proposed arrangement of the active center in the monolayer.

Although these observed shifts were very small, the same tendency was obtained from the data, which might imply ECT from Fe to Pd, or to Ru in the monolayer. The similar ECT might also occur from Ru to Pd in the trimetallic nanosheet (Table S8†). This sequential electronic charge transfer can be illustrated, as shown in Scheme 2.

This sequence of electron transferring also could be supported and explained by the individual component function for boosting the activity. From the results of the catalytic activity for GO@H-Pd/Fe/Ru obtained above (Table S2†), GO@H-Pd/Fe exhibited a higher catalytic activity compared with that of GO@H-Pd/Ru, indicating that Fe could improve the activity more than Ru. However, if the combination of GO@H-Pd/Fe/Ru was made, it exhibited its effect to the maximum and achieved a much higher improvement in the catalytic performance, in which the activity of GO@H-Pd/Fe/Ru was two or five times higher than those of GO@H-Pd/Fe and GO@H-Pd/Ru. Thus, the arrangement of these three metallic complexes in the monolayer was presumed as Fe to Pd to Ru, in which a metal Pd(ii) was juxtaposed with one Fe(iii) and one Ru(iii) that had a strong tendency to adsorb phenyl boronic acid (Scheme 2). It exhibited this intimate contact with the Fe and Ru to the maximum, and when combined with a high activity of Fe–Ru–Pd–Fe, achieved an improvement in the catalytic properties.^33^ The results also indicated that the Pd0–Ru(iii)–Pd(ii)–Fe(iii) ensemble sites were required for the catalytic process, in which the real active sites were not only trace Pd_0_, but an ensemble cooperation with other metallic sites, including a ligand effect and electronic effect. It was possible for making either sites active towards cross-coupling catalysis.^3,92^ The XPS data indicated that the electronic structure of Pd was modified by Fe and Ru, which would result only if Pd were in intimate contact with Fe or Ru (Scheme 2).

**Fig. 12:**
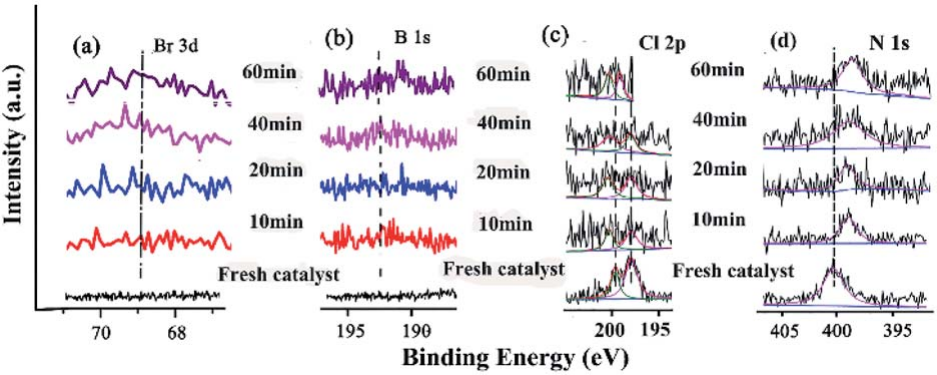
XPS of the catalyst and recycled catalyst: (a) Br 3d, (b) B 1s, (c) Cl 2p, (d) N 1s.

By comparing the different reaction times and the changes of B 1s, Br 3p, and N 1s in the catalytic process, it was helpful for elucidating the catalytic mechanism (Fig. 12). These three elements showed a trend of enhancement from the initial non-characteristic peak to the characteristic peak with the extension of the reaction time. This indicated that there was a substrate adsorption process in the catalytic process.

The characterization of the changes of elements on the surface was very important for investigating the catalytic mechanism (Fig. 12). The Br 3d and B 1s appeared with increasing time (Fig. 12a and b), indicating that the substrates containing these elements were adsorbed and reacted at some active site. The Cl 2p peaks at 198.08 eV and 199.82 eV gradually shifted to higher BE during catalysis (Fig. 12c and Table S14†), meaning that Cl coordinated with Pd and Pd^2+^. In the case of N 1s (the C=N double bond) at 401.51 eV, the peak shifted to lower BE during catalysis (Fig. 12d and Table S14†), indicating that with the Schiff-based Pd(ii) as a released precursor, the active Pd(0) and Schiff-base group in the ligand could stabilize and prevent the Pd(0) from aggregating and leaching during the reaction.

**Scheme 3:**
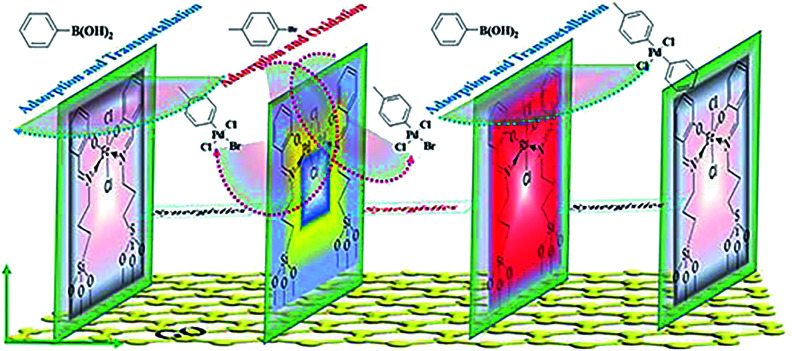
Plausible catalytic mechanism in the Suzuki coupling reaction.

Based on the results of the Raman, SEM, TEM, and XPS analysis, the catalytic mechanism could be proposed as depicted in Scheme 3. The approach outlined here offers opportunities for both fundamental research and application, which delineated the underlying mechanistic steps on the surfaces. It was assumed that the trimetallic complexes were a pre-catalyst with certain types of active sites that had their functions in order to achieve a surface-catalyzed reaction, in which an active electron-rich Fe–Pd–Ru–Fe center was formed by synergetic between Pd, Fe and Ru. There is a strong correlation between the catalytic activity and adjustable certain substrate adsorption capacity in the vicinity of the Fe–Pd–Ru–Fe center. Pd adsorbed *p*-bromotoluene to form an oxidation intermediate, which reacted with phenyl boronic acid adsorbed on the neighboring Ru or Fe to yield the transmetallic intermediate (Scheme 3). The presence of the Ru or Fe species around Pd on the surface improved the substrate adsorption, leading to a high efficiency.^93^

## 4. Conclusions

In summary, the trimetallic catalytic nanosheet immobilized on GO was fabricated by self-assembly. GO@H-Fe_95.73_Pd_4.26_Ru_0.01_ showed better stability and recyclability (at least 10 times with higher activity). The heterogeneous catalytic mechanism was demonstrated by kinetics, hot filtration, poisoning tests and *in situ* ReactIR, and the effect of the supports was also investigated in detail. The investigation of the deactivation mechanism of GO@H-Fe_95.73_Pd_4.26_Ru_0.01_ showed that the deactivation was related to the agglomeration of the active species, leaching of the active species, changes of the catalyst structure, and active sites covered by residues of elements. The synergistic catalytic mechanism over the hetero-trimetallic nano-monolayer based on the results obtained above may be the following main aspects: (1) enhanced activity results from the ordered orientation of the catalytic nano-monolayer upon introducing third metal. (2) Electronic effect among these three different metals and the structure of the ligand enhanced the catalytic activity. (3) Among this trimetallic (Pd, Fe and Ru) catalytic nanosheet, although Fe is less active, the doping of Fe or Ru to Pd can effectively enhance the entire activity of the trimetallic nanosheet. The presence of Fe made Pd more negative and increased the stability of the catalyst. The Ru metal adjacent to Pd helps to activate the substrate molecules adsorbed on Ru, and made it easy for the trans-metalation step.

## Conflicts of interest

There are no conflicts to declare.

## Supplementary Material

RA-011-D0RA09347E-s001
