# Supplementary material for: A New ternary organometallic Pd(ii)/Fe(iii)/Ru(iii) self-assembly monolayer: the essential ensemble synergistic for improving catalytic activity
Source: RSC Adv. 2021 Jan 4;11(3):1250–60. doi: 10.1039/d0ra09347e (PMC8693531; doi:10.1039/d0ra09347e)
Supplement: RA-011-D0RA09347E-s001 [file RA-011-D0RA09347E-s001.pdf]

Electronic Supplementary Material (ESI) for RSC Advances.
This journal is © The Royal Society of Chemistry 2020

Supporting Information

A New Ternary Organometallic Pd(II)/Fe(III)/Ru(III) Self-assembly Monolayer: The Essential Ensemble Synergistic for Improving Catalytic Activity

Ruirui Ren[a], Pingping Huang[a], Wuduo Zhao[a], Tiesheng Li[a*], Minghua Liu[b,c*] ,Yangjie Wu[a*]

[a]College of Chemistry and Molecular Engineering, Zhengzhou University, Kexuedadao 100, Zhengzhou, 450001, P. R. China

[b] Henan Institute of Advanced Technology, Zhengzhou University, Kexuedadao 100, Zhengzhou 450001, Henan Province, P.R. China.

[c]Beijing National Laboratory for Molecular Science, Institute of Chemistry, Chinese Academy of Sciences, Zhongguancun North First Street 2, Beijing 100190, P. R China.

*Corresponding Author: Tiesheng Li, E-mail: lts34@zzu.edu.cn.

Fax :(+)86-371-67766667

1. Experimental section

1.1 General methods

Chemical regents were obtained from commercial sources. Solvents were distilled using appropriate drying agents under nitrogen. X-Ray diffraction (XRD) was performed on a PAN analytical X-Pert PRO instrument. Fourier transform infrared (FTIR) spectroscopy was carried out on a BRUKER TENSOR FTIR spectrometer using KBr pellets. Raman spectra were measured with a Thermo Scientific DXR Raman microscope with an excitation laser wavelength of 532 nm. XPS data were obtained using an ESCALab220i-XL electron spectrometer from VG Scientific with 300 W Al Kα radiation. Scanning electron microscopy (SEM) images were recorded using a Hitachi S-4800 system. Transmission electron microscopy (TEM) images were recorded using a JEM-2100F transmission electron microscope operating at 200 kV. The Pd and Co content in the catalysts before and after the cross-coupling reactions was measured by inductively coupled plasma atomic emission spectroscopy (ICP-AES) with an ICAP 6000 Series (Thermo Scientific). The sample treatments were as follows: the sample was broken down with nitrolysis, and then residual solid was dissolved with 2 M hydrochloric acid and transferred into a 10 mL volumetric flask to fix its quantity in water. ^{1}H NMR and^{13} C NMR spectra were recorded on a Bruker Advance III 400 MHz spectrometer in $CDCl_3$ with tetramethylsilane as an internal standard. A Vertex 70 V spectrometer (Bruker Optik, Ettlingen, Germany) at 293 K, with a spectral resolution of 4 cm^{-1} and a scanner

velocity of 10 kHz was used for monitoring the coupling reaction of 4-bromotoluene with phenylboronic acid. AFM images were measured on AIST-NT, AC(tapping) mode.

1.2. Fabrication and characterization of **GO@H-Pd/Fe/Ru** monolayer

Preparation of **GO@H-Pd/Fe/Ru** monolayer was carried out according the literature reported [16].

1.2.1 Preparation of the amino modified graphene oxide (denoted as **H-GO**)

Firstly, synthesis of nitrogenous ligands (salen). o-hydroxybenzaldehyde 0.17mL (1.6mmol) and 3-Amino-propyltriethoxysilane (APTES) 0.38mL (1.6mmol) were dissolved in 30mL absolute ethanol and refluxed at 70°C for 12h. After the solvent was removed using a rotary evaporator. And it was dried in a vacuum oven at 40°C for 12h. 97% yield was obtained. ^{1}H NMR (400MHz, $CDCl_3$, ppm): δ 13.81 (s,1H), 8.33 (s,1H), 7.31 (d,1H), 7.27 (t,1H), 6.96 (d,1H), 6.87 (t,1H), 3.84 (q,6H), 3.60 (t,2H), 1.86 (m,2H), 1.24 (t,9H),0.70 (t,2H). ^{13}C NMR (400MHz, $CDCl_3$, ppm): δ 164.51, 161.14, 131.78, 130.85, 118.55, 118.11, 116.78, 61.78, 58.11, 24.11, 18.04, 7.70. see additive.

Ref. *Mungse H P, Verma S, Kumar N, et al. Grafting of oxo-vanadium Schiff base on graphene nanosheets and its catalytic activity for the oxidation of alcohols[J]. Journal of materials chemistry, 2012, 22(12): 5427-5433.*

Then, **GO** (1g) was dispersed in toluene (100 mL) by ultrasonic processing about 2h. Then, nitrogen group (1.6 mmol) was added to round-bottom flask and refluxed at 80°C for 24h. The product was separated by filtration and washed with appropriate solvent three times. Nitrogen group modificatory graphene oxide (denoted as **H-GO**) was dried in a vacuum oven at 40°C for 12h.

1.2.2 Preparation of **GO@H-Pd/Fe/Ru**

H-GO (1g) was dissolved in anhydrous methyl alcohol (30mL) sonicated for 0.5h. Then, Different ratio of $Li_2PdCl_4/FeCl_3{\cdot}6H_2O/RuCl_3$ mixture (total 1.6 mmol) was added to mixture with continuous stirring at 40°C for 24h. The product was separated by filtration and washed with appropriate solvent three times. **GO@H-Pd/Fe/Ru** was obtained by drying in a vacuum oven at 40°C for 12h.

1.3 Procedure for Suzuki reaction and recycling

Si@H-Pd/Fe/Ru(1mg), base, and reactant were added to a 10 mL round-bottom flask with 4 mL solvent. The reaction was carried out in an oil bath at 80 °C for a certain time. The catalysts were separated from the reaction mixture. For the recycling experiments, the reactions were carried out under the above conditions. After each run, the used catalyst was recovered from the reaction mixture and reused in sequential runs after washing with ethyl acetate, methyl alcohol, and water three times.

1.4 ReactIR dynamic analysis

ReactIR analysis are carried out as follow: A 10 mL two-neck flask with a magnetic stirrer bar was equipped with the ReactIR probe to monitor the reaction in a

70 °C oil bath. 4 mL solvent, 1.0 mmol reactant, and 2.0 mmol K_2CO_3 were added to the two-neck flask and dispersed by rapid ultrasonic processing. After that, a background spectrum was recorded. Then scans were performed in the time resolved spectroscopy (TRS) mode with 60 s intervals for 30 min.

2. Characterization of the GO@H-Pd/Fe/Ru monolayer

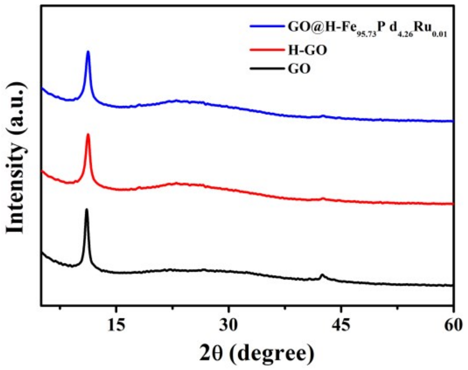

Figure S1. XRD spectra of **GO**, **H-GO** and **GO@H-$Fe_{95.73}Pd_{4.26}Ru_{0.01}$**.

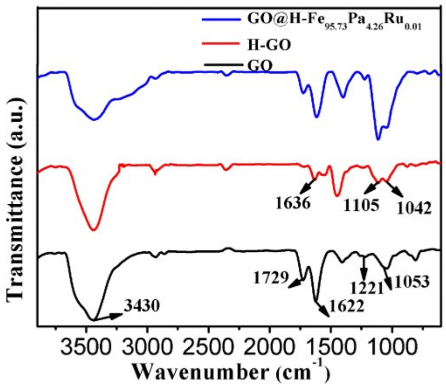

Figure S2. FT-IR of **GO**, **H-GO** and **GO@H-$Fe_{95.73}Pa_{4.26}Ru_{0.01}$**.

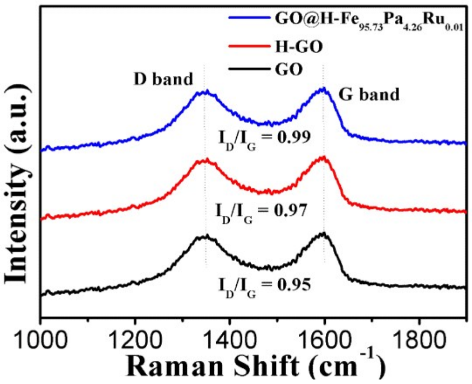

Figure S3. Raman spectra of **GO**, **H-GO** and **GO@$Fe_{95.73}Pa_{4.26}Ru_{0.01}$**.

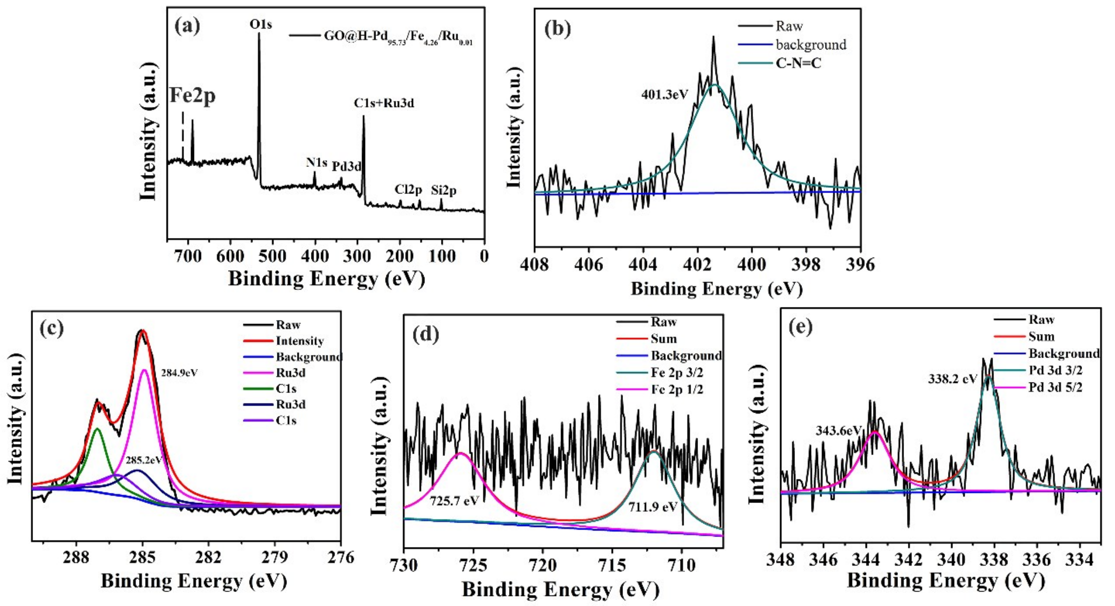

Figure S4. (a) Full range XPS spectra of **GO@H-$Fe_{95.73}Pd_{4.26}Ru_{0.01}$**.; (b) N 1s; (c) Ru 3d +C 1s; (d) Fe 2p; (e) Pd 3d.

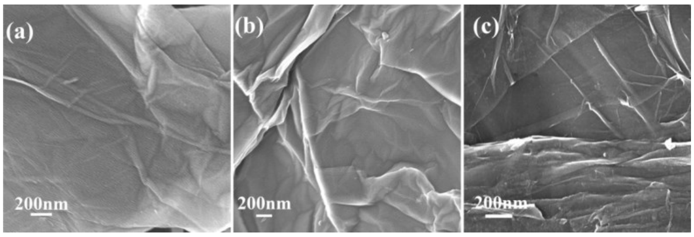

Figure S5. SEM images of (a) **GO** (b) **H-GO** (c) **GO@H-$Fe_{95.73}Pd_{4.26}Ru_{0.01}$**.

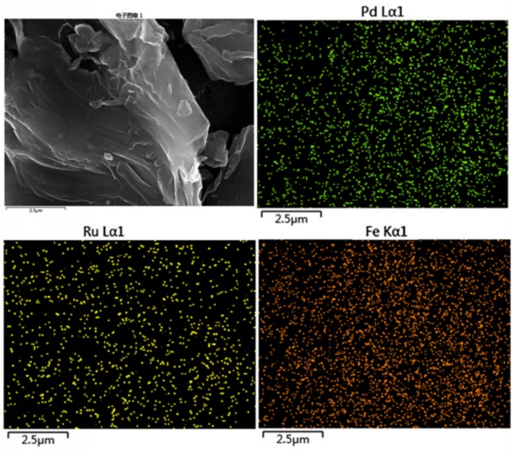

Figure S6. Element mapping images of **GO@H-$Fe_{95.73}Pd_{4.26}Ru_{0.01}$**.

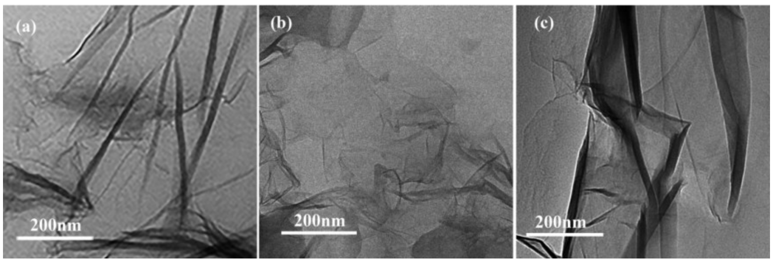

Figure 7. TEM images of (a) **GO** (b) **H-GO** (c) **GO@H-Fe$_{95.73}$Pd$_{4.26}$Ru$_{0.01}$**.

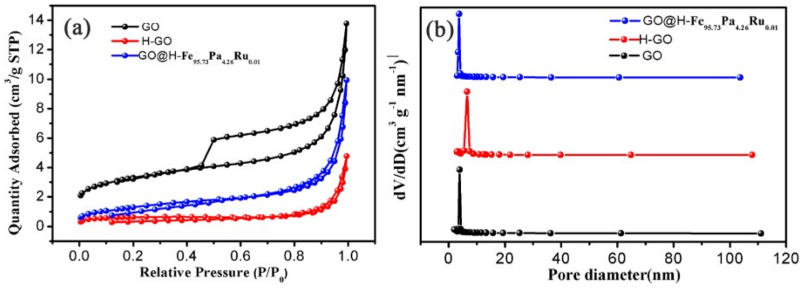

Figure S8. (a) N_2 adsorption-desorption isotherms, (b) the corresponding pore size distribution of **GO**, **H-GO** and **GO@H-Fe$_{95.73}$Pd$_{4.26}$Ru$_{0.01}$**.

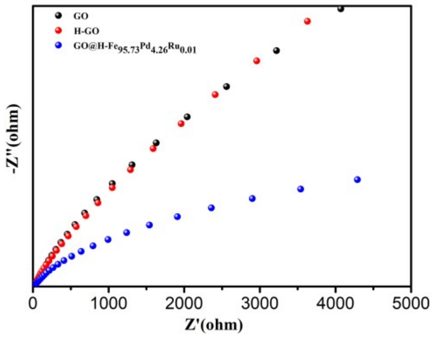

Figure S9. Electrochemical impedance spectra (EIS) of the catalyst on the Ni foam for **GO**, **H-GO** and **GO@H-Fe$_{95.73}$Pd$_{4.26}$Ru$_{0.01}$.**

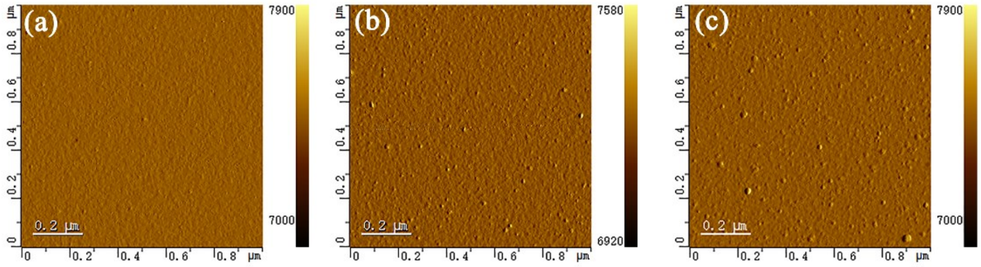

Figure S10 AFM topographic images of the different functionalised Si surfaces (a) **Si@OH** (hydrophilic treatment), (b) **Si@H** (Salicylaldehyde), (c) **Si@H-PdFeRu**,

3. Investigation on catalytic properties of GO@H-Fe$_{95.73}$Pd$_{4.26}$Ru$_{0.01}$ monolayer

3.1 Optimization

Table S1 Optimization of Suzuki reaction conditions catalyzed by **GO@H-Fe$_{95.73}$Pd$_{4.26}$Ru$_{0.01}$.**

Entry	Base	Solvent	Time(h)	T(℃)	Isolated yield(%)	TON	TOF(h^{-1})
1	Na_2CO_3	H_2O	1	80	50	8710	8710
2	Na_2CO_3	EtOH	1	80	3	522	522
3	Na_2CO_3	DMF	1	80	11	1916	1916
4	Na_2CO_3	MeOH	1	80	13	2264	2264
5	Na_2CO_3	H_2O:EtOH(1:1)	1	80	99	17247	17247
6	Na_2CO_3	H_2O:EtOH(2:1)	1	80	99	17247	17247
7	Na_2CO_3	H_2O:EtOH(3:1)	1	80	99	17247	17247
8	Na_2CO_3	H_2O:EtOH(4:1)	1	80	67	11672	11672

9	Na_2CO_3	Toluene	1	80	trace	-	-
10	K_2CO_3	H_2O:EtOH(3:1)	1	80	99	17247	17247
11	K_3PO_4	H_2O:EtOH(3:1)	1	80	87	15156	15156
12	NaOH	H_2O:EtOH(3:1)	1	80	97	16898	16898
13	NaOAc	H_2O:EtOH(3:1)	1	80	10	1742	1742
14	$NaHCO_3$	H_2O:EtOH(3:1)	1	80	49	8536	8536
15	Et_3N	H_2O:EtOH(3:1)	1	80	7	1219	1219
16	Na_2CO_3	H_2O:EtOH(3:1)	0.5	80	82	28571	14285
17	Na_2CO_3	H_2O:EtOH(3:1)	1	70	76	13240	13240
18	Na_2CO_3	H_2O:EtOH(3:1)	1	60	8	1393	1393
19	Na_2CO_3	H_2O:EtOH(3:1)	1	80	77[b]	26892	26892

[a] Reaction condition: $PhB(OH)_2$ (0.5 mmol), 4-bromotoluene (0.5 mmol), Base (1 mmol), **GO@H-$Fe_{95.73}Pd_{4.26}Ru_{0.01}$** 1 mg , solvent (4 mL) at 80 ℃. [b] Reaction condition: $PhB(OH)_2$ (1mmol), 4-bromotoluene 1 mmol), Base (2mmol), **GO@H-$Fe_{95.73}Pd_{4.26}Ru_{0.01}$** 1 mg , solvent (4 mL) at 80℃.

3.2 Influences of the ratio of Fe /Pd /Ru on catalytic performance

Table S2 Influences of the ratio of Fe/Pd/Ru on the catalytic performance.

Entry	Catalyst	Pd (molg^{-1})	Fe (mol·g^{-1})	Ru (mol·g-1)	Time (h)	Isolated yield(%)	TON	TOF(h^{-1})
1	GO@H-Ru	-	-	9.04×10^{-6}	1	2	-	-
2	GO@H-Pd	6.03×10^{-5}	-	-	1	99	8291	8291
3	GO@H-Fe	-	2.05×10^{-4}	-	1	trace	-	-
4	GO@H-PdFe	6.81×10^{-5}	2.34×10^{-4}	-	1	99	7268	7268
5	GO@H-FeRu	-	1.58×10^{-4}	9.32×10^{-6}	1	3	-	-
6	GO@H-PdRu	1.49×10^{-4}	-	1.01×10^{-6}	1	99	3300	3300
7	GO@H-$Fe_{91.74}Pd_{8.16}Ru_{0.1}$	5.60×10^{-5}	8.34×10^{-5}	2.31×10^{-7}	1	99	8839	8839
8	GO@H-$Fe_{91.83}Pd_{8.16}Ru_{0.01}$	4.63×10^{-5}	8.18×10^{-5}	9.50×10^{-7}	1	99	10691	10691
9	GO@H-$Fe_{95.73}Pd_{4.26}Ru_{0.01}$	2.87×10^{-5}	7.98×10^{-5}	8.70×10^{-7}	1	99	17247	17247
10	GO@H-$Fe_{90.82}Pd_{2.17}Ru_{0.01}$	1.75×10^{-5}	8.84×10^{-5}	5.10×10^{-7}	1	76	21714	21714

[a] Reaction condition: $PhB(OH)_2$ (0.5 mmol), 4-bromotoluene (0.5 mmol), Na_2CO_3(1 mmol), **GO@H-$Fe_{95.73}Pd_{4.26}Ru_{0.01}$** 1 mg, solvent (4 mL) at 80℃ for 1h.

3.3 Catalytic properties of **GO@H-$Fe_{95.73}Pd_{4.26}Ru_{0.01}$**

Table S3 Influences of supports on the catalytic performance.

Entry	Catalyst	Pd loading (mol·g^{-1})	Yield (%)	TOF(h^{-1})
1	GO	-	0[b]	
2	H-GO	-	0[c]	
3	Li_2PdCl_4/$FeCl_3$.6H_2O/$RuCl_3$·XH_2O	2.87×10^{-5}	84[d]	14634
4	H+Li_2PdCl_4/$FeCl_3$.6H_2O/$RuCl_3$·XH_2O	2.87×10^{-5}	42	7317
5	GO+Li_2PdCl_4/$FeCl_3$.6H_2O/$RuCl_3$·XH_2O	2.87×10^{-5}	64	11149
6	H-GO+Li_2PdCl_4/$FeCl_3$.6H_2O/$RuCl_3$·XH_2O	2.87×10^{-5}	49	8536
7	GO@H-$Fe_{95.73}Pd_{4.26}Ru_{0.01}$	2.87×10^{-5}	99[e]	17247
8	Silica@H-$Fe_{95.73}Pd_{4.26}Ru_{0.01}$	5.34×10^{-4}	76[f]	700

[a] Reaction condition: $PhB(OH)_2$ (0.5mmol), 4-bromotoluene (0.5 mmol), Na_2CO_3 1 mmol), catalyst 1 mg, solvent (25% aqueous alcohol 4 mL) at 80℃ for 1 h .[b] GO 1mg. [c] H-GO 1mg. [d] Li_2PdCl_4 0.0000287 mmol and $FeCl_3$.6H_2O and $RuCl_3$·XH_2O. [e] GO@H-$Fe_{95.73}Pd_{4.26}Ru_{0.01}$1mg. [f] Silica@H- $Fe_{95.73}Pd_{4.26}Ru_{0.01}$ 1 mg.

Table S4 Comparison for Suzuki coupling reaction catalyzed by different catalysts

Entry	Catalyst	Reaction conditions	X	Yield (%)	TOF (h^{-1})	Run	Ref
**This work**	GO@H-$Fe_{95.73}Pd_{4.26}Ru_{0.01}$ (0.0057mol% Pd)	Na_2CO_3, EtOH:H_2O, 1h,80°C	Br	99	17247	10	-
**2**	Pd/Fe_3O_4/s-G (0.15mol% Pd)	K_2CO_3, EtOH:H_2O, 30min,80°C	Br	97	1293	10	58
**3**	Pd/Fe_3O_4/r-GO (0.36mol% Pd)	K_2CO_3, H_2O, 2h,80°C	Cl	85	118	8	57
**4**	GO-2N-Pd(II) (0.5mol% Pd)	K_2CO_3, EtOH, 4h,80°C	Br	77	39	6	45
**5**	Fe_3O_4@Void@mSiO_2-Pd(0) (0.142 mol% Pd)	K_2CO_3, IPA, 10h,80°C	Br	99	70	7	14
**6**	GO@H -$Pd_{0.10}Fe_{0.90}$ (0.0106 mol%Pd)	Na_2CO_3, EtOH:H_2O, 2h,70°C	Br	86	8074	9	16
**7**	Pd-Fe/BNNS (0.02 mol%Pd)	$(CH_3)_3COK$, EtOH:H_2O, 0.5h, Reflux	Br	99	9900	6	29
**8**	Pd/$NiFe_2O_4$ (6 mol%Pd)	Na_2CO_3, DMF:H_2O, 0.08h,90°C	I	97	196	5	9
**9**	Starch supported Pd(II catalyst) (4×10^{-3}mol% Pd)	K_2CO_3/, free solvent media,50°C, 0.08 h under MW	Br	100	312500	10	11
**10**	$PdCu_3$ (0.697 mol% Pd)	NaOH, H_2O, 1h,75°C	Br	97.6	140	4	80
**11**	Pdtnp (1:1:1) (0.167 mol%Pd)	CH3COONa, DMF:H_2O, 12h,100°C	I	99	49	3	30
**12**	Fe@Pd/C (0.5 mol%Pd)	K_2CO_3, H_2O, 0.5h,70°C	I	100	400	5	76
**13**	Pd-$CoFe_2O_4$ NPs (1.6 mol% Pd)	Na_2CO_3, EtOH 12h, Reflux	Br	79	4	4	10
**14**	$Pd_{0.5}Ru_{0.5}$–PVP NPs (0.08 mol% Pd)	K_2CO_3, DMA: H_2O, 100°C, 0.08 h	Br	96	15000	3	3
**15**	Pd(0)/MCoS-1 (20 mg catalyst)	K_2CO_3, H_2O, 5h,70°C	Br	96	96	6	75
**16**	GO-NH_2-Pd (1 mol% Pd)	K_2CO_3, EtOH:H_2O, 4h,60°C	Br	73	18	10	64
**17**	GO-NHC-Pd (0.25 mol% Pd)	K_2CO_3, EtOH:H_2O, 3h,80°C	Br	93	124	6	65
**18**	Pd/CNT-SiC (0.06 mol% Pd)	K3PO4, EtOH :H_2O, 1h,60°C	I	98	1750	6	59
**19**	RuII –PdII–RuII type complexes(1.5 Pd mol%	Cs_2CO_3, DMF: H_2O, 80°C,2h	Br	95	316	-	27
**20**	Ni/Pd core−shell NPs/graphene	K_2CO_3, DMF:H_2O, 0.5h,110°C	Br	78	208	5	26

(0.75 mol% Pd)

Table S5 Suzuki coupling reaction of aryl halides with different aryl-boronic acids.

Entry	Ar-X / Ar'-B(OH)$_2$ / Product	Yield(%)
1	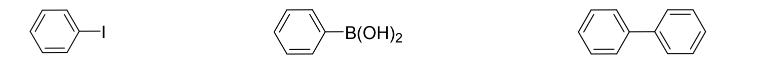	99
2	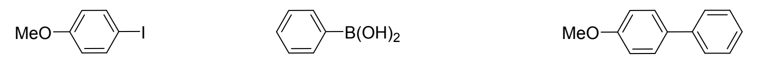	99
3	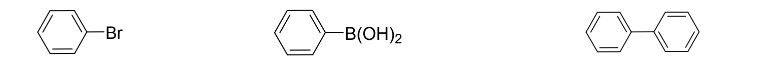	99
4	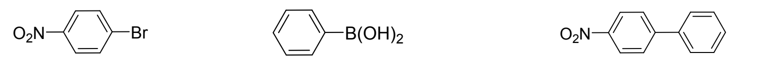	99
5	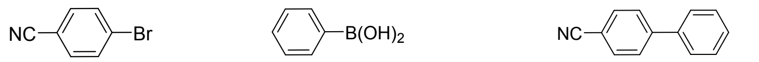	99
6	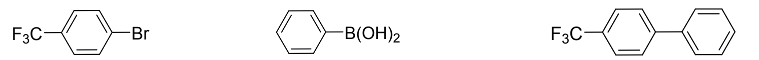	97
7	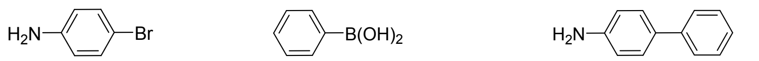	99
8	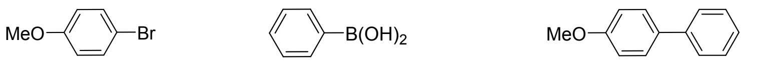	98
9	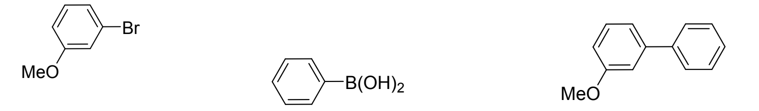	97
10	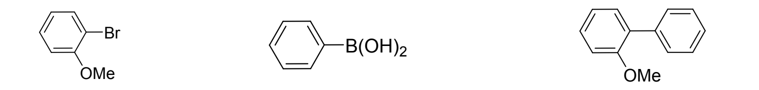	86
11	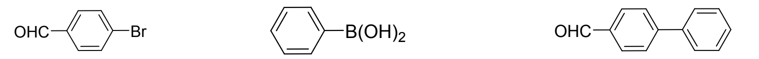	99
12	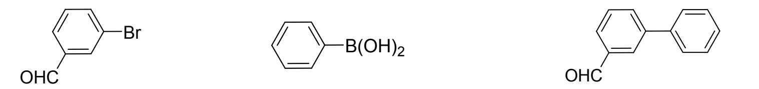	99
13	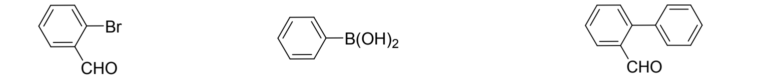	76
14	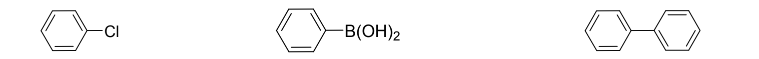	6
15	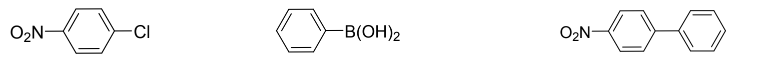	8
16	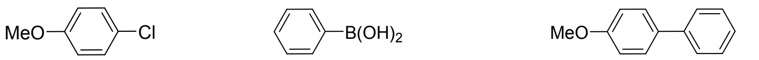	5
17	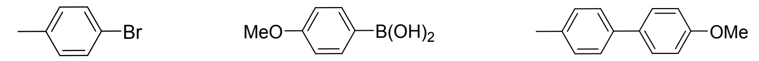	98
18	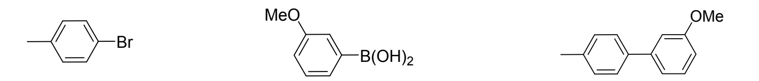	92
19	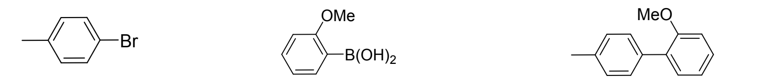	83

20	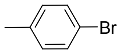	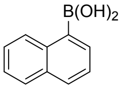	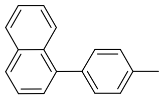	83
21	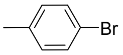	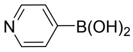	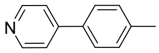	trace
22	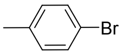	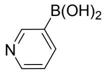	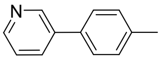	7

[a] Reaction condition: $PhB(OH)_2$ derivatives (0.5mmol), 4-bromotoluene derivatives (0.5 mmol), Na_2CO_3 1 mmol), catalyst 1 mg, solvent (25% aqueous alcohol 4 mL) at 80℃ for 1 h.

4.The deactivation mechanism

Table S6 Deconvolution data for Ru 3d + C 1s

Recycle times	Ru 3d + C1s Binding Energy(eV)			
0	284.95	285.15	285.95	287.05
1st	284.64	284.91	286.29	286.84
4th	284.52	285.48	286.69	288.67
8th	284.67	285.32	286.54	288.65
10th	284.64	285.55	286.55	288.44

Table S7 Deconvolution data for Pd 3d and Fe 2p

Recycle times	Pd 3d Binding Energy (eV)				Fe 2p Binding Energy (eV)			
0	343.60		338.20		711.90		725.70	
1st	343.60	340.88	338.35	335.49	712.52		726.45	
4th	343.47	340.88	338.38	335.77	712.53	719.49	725.70	735.60
8th	343.49	340.87	338.28	335.49	712.68	719.64	725.90	736.76
10th	343.50	340.82	338.37	335.79	712.54	719.83	725.44	735.33

Table S8 Ratio of Pd^0/Pd^{2+} during recycling

Recycle times	Ratio of Pd^0/Pd^{2+}
0	-
1st	0.92
4th	1.02
8th	0.94
10th	0.98

Table S9 Deconvolution data for Cl 3p and N 1s

Recycle times	Cl 2p BE(eV)		N 1s BE (eV)
0	198.08	199.82	401.51
1st	199.35	200.56	399.94
4th	198.68	200.84	399.87
8th	198.94	200.82	399.35
10th	200.02	202.26	399.86

5.Investigation on catalytic mechanism

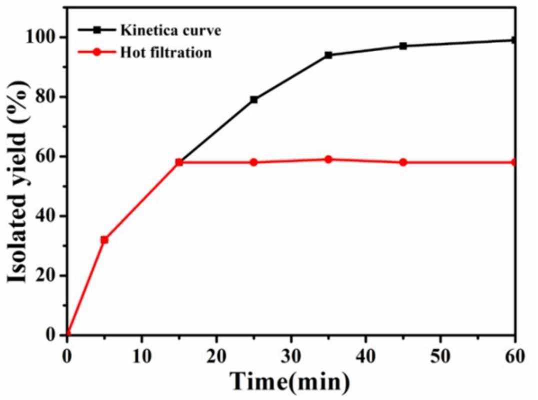

Figure S11 Hot filtration experiment.

Table S10. Poisoning experiments of **GO@H-$Fe_{95.73}Pd_{4.26}Ru_{0.01}$**

Entry	Poisoning additive	Isolated yield (%)
1	Hg	14[a]
2	2,2'-Dipyridyl	trace[b]
3	Thiophene	6[c]

[a] Reaction condition: $PhB(OH)_2$ (0.25 mmol), 4-bromotoluene (0.5 mmol), Na_2CO_3 (1 mmol), GO@H-$Fe_{95.73}Pd_{4.26}Ru_{0.01}$, solvent (4 m L) at 80°C for 1 h . [b] One drop of Hg. [c] 0.5 equiv of 2,2'-Dipyridyl (per metal atom) . [d] 0.5 equiv of thiophene (per metal atom).

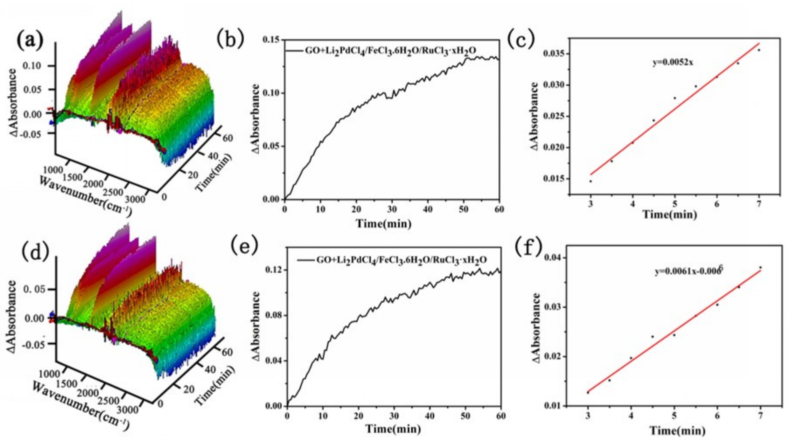

Figure S12. ReactIR plots over time for the formation of 4-phenyltoluene by Suzuki coupling reaction (a) 3D map catalyzed by GO+$Li_2PdCl_4/FeCl_3.6H_2O/RuCl_3 \cdot xH_2O$ at 80 ℃, (b) and (c) Kinetic analysis of the catalytic reaction of GO+$Li_2PdCl_4/FeCl_3.6H_2O/RuCl_3 \cdot xH_2O$ using the band of 754 cm^{-1} at 80 ℃, (d) 3D map catalyzed by GO+$Li_2PdCl_4/FeCl_3.6H_2O/RuCl_3 \cdot xH_2O$ at 65 ℃, (e) and (f) Kinetic analysis of the catalytic reaction of GO+$Li_2PdCl_4/FeCl_3.6H_2O/RuCl_3 \cdot xH_2O$ using the band of 754 cm^{-1} at 65 ℃.

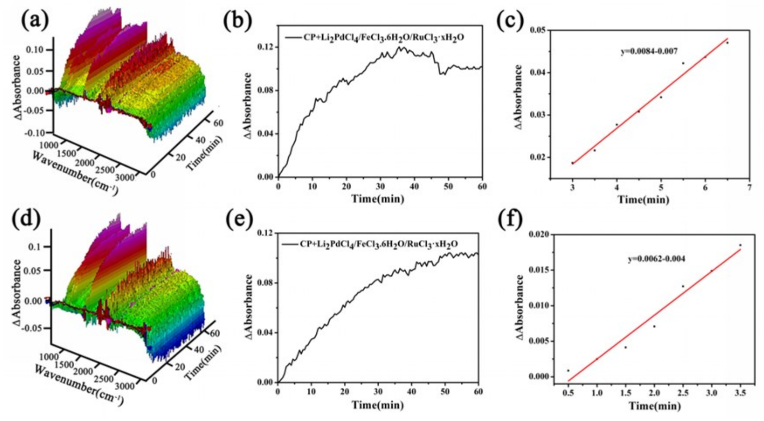

Figure S13. ReactIR plots over time for the formation of 4-phenyltoluene by Suzuki coupling reaction (a) 3D map catalyzed by CP+$Li_2PdCl_4/FeCl_3.6H_2O/RuCl_3 \cdot xH_2O$ at 80 ℃, (b) and (c) Kinetic analysis of the catalytic reaction of CP+$Li_2PdCl_4/FeCl_3.6H_2O/RuCl_3 \cdot xH_2O$ using the band of 754 cm^{-1} at 80 ℃, (d) 3D map catalyzed by CP+$Li_2PdCl_4/FeCl_3.6H_2O/RuCl_3 \cdot xH_2O$ at 65 ℃, (e) and (f) Kinetic analysis of the catalytic reaction of CP+$Li_2PdCl_4/FeCl_3.6H_2O/RuCl_3 \cdot xH_2O$ using the band of 754 cm^{-1} at 65 ℃.

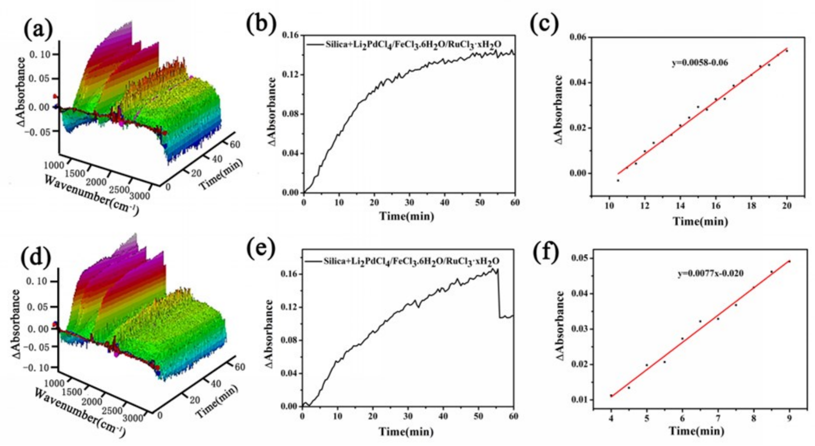

Figure S14. ReactIR plots over time for the formation of 4-phenyltoluene by Suzuki coupling reaction (a) 3D map catalyzed by Silica+$Li_2PdCl_4/FeCl_3.6H_2O/RuCl_3 \cdot xH_2O$ at 80 ℃, (b) and (c) Kinetic analysis of the catalytic reaction of Silica+$Li_2PdCl_4/FeCl_3.6H_2O/RuCl_3 \cdot xH_2O$ using the band of 754 cm^{-1} at 80 ℃, (d) 3D map catalyzed by Silica+$Li_2PdCl_4/FeCl_3.6H_2O/RuCl_3 \cdot xH_2O$ at 65 ℃, (e) and (f) Kinetic analysis of the catalytic reaction of Silica+$Li_2PdCl_4/FeCl_3.6H_2O/RuCl_3 \cdot xH_2O$ using the band of 754 cm^{-1} at 65 ℃.

Table S11 Results of Kinetic analysis in homogeneous system with different support added

Catalyst	T(℃)	Rate constants	Apparent activation energy (kJ/mol)
GO+Li_2PdCl_4/$FeCl_3.6H_2O$/$RuCl_3·xH_2O$	80	k_1=0.0052	10.6
	65	k_2=0.0061	
CP+Li_2PdCl_4/$FeCl_3.6H_2O$/$RuCl_3·xH_2O$	80	k_1=0.0085	20.9
	65	k_2=0.0062	
Silica+Li_2PdCl_4/$FeCl_3.6H_2O$/$RuCl_3·xH_2O$	80	k_1=0.0058	10.9
	65	k_2=0.0077	

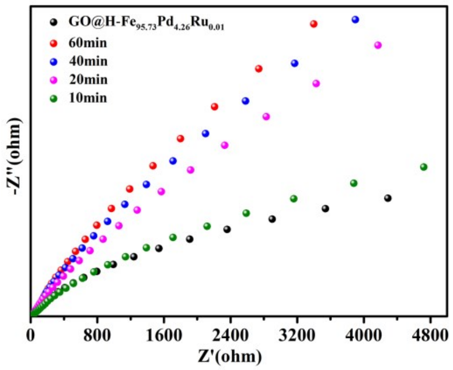

Figure S15.Electrochemical impedance spectra (EIS) of **GO@H-$Fe_{95.73}Pd_{4.26}Ru_{0.01}$**.

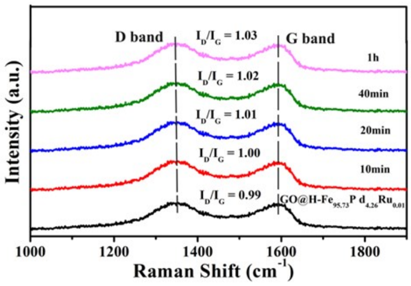

Figure S16. Raman spectra of **GO@H-$Fe_{95.73}Pa_{4.26}Ru_{0.01}$** in catalytic process.

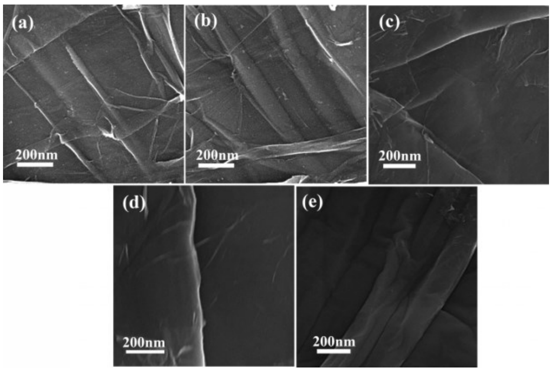

Figure S17. SEM images of the process of catalyst and reused catalyst (a) 0 min, (b) 10 min, (c) 20 min, (d) 40 min, (e) 60min

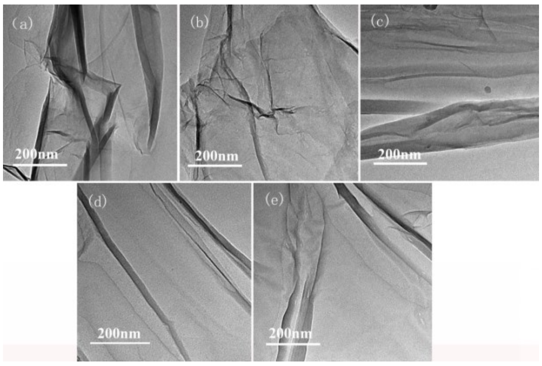

Figure S18. TEM images of the process of catalyst and reused catalyst (a) 0 min, (b) 10 min, (c) 20 min, (d) 40 min, (e) 60min

Table S12 Deconvolution data for Ru3d+C1s during catalysis

Catalytic time/min	Ru 3d + C1s Binding Energy(eV)			
0	284.95	285.15	285.95	287.05
10	284.56	284.77	285.82	286.69
20	284.79	284.80	285.87	286.49
40	284.51	284.69	285.26	286.47
60	284.64	284.91	286.29	286.84

Table S13 Deconvolution data for Pd 3d and Fe 2p during catalysis

Catalytic time/min	Pd 3d Binding Energy (eV)				Fe 2p Binding Energy (eV)			
0	343.60		338.20		711.90		725.70	
10	343.61		338.35		712.59		725.45	738.79
20	343.61	340.84	338.37	335.39	712.65		725.87	736.74
40	343.61	340.84	338.35	335.39	712.43	718.65	725.69	736.73
60	343.60	340.88	338.35	335.49	712.52		726.45	

Table S14 Deconvolution data for Cl 2p and N 1s in different catalytic time

No.	Time(min)	Cl 2p Binding Energy (eV)		N 1s Binding Energy (eV)
1	0	198.08	199.82	401.51
2	10	198.12	200.46	400.06
3	20	198.14	200.63	400.26
4	40	198.19	200.62	399.96
5	60	199.35	200.56	399.94

6. Additive

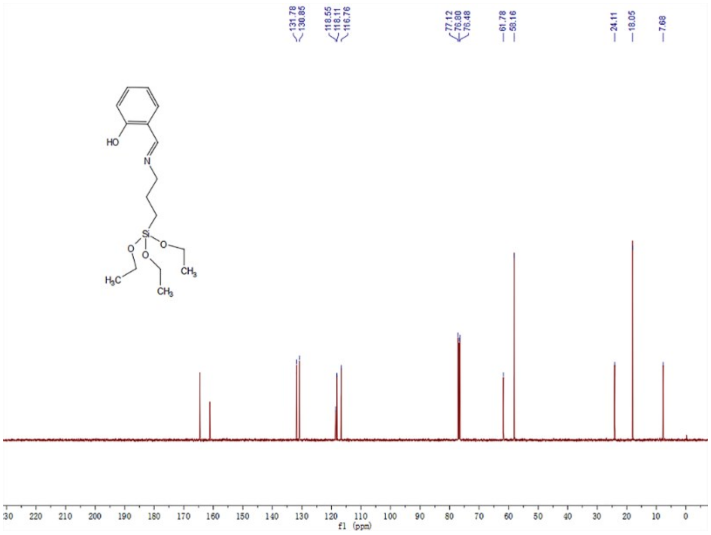

^{1}H NMR of nitrogenous ligands (salen)

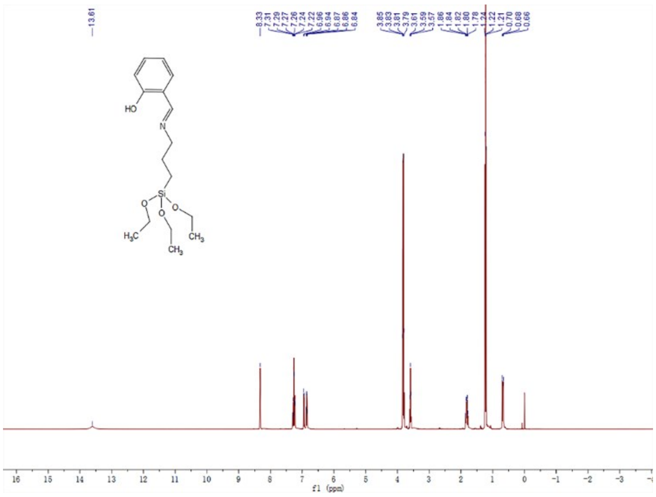

^{13}C NMR of nitrogenous ligands (salen)
